# A framework for the continuous monitoring of person-centred hospital care: validation of a checklist for participatory service improvement

**DOI:** 10.1186/s13690-024-01410-5

**Published:** 2025-01-14

**Authors:** F. Cardinali, S. Carzaniga, L. Martini, M. T. Loiudice, Fabrizio Carinci

**Affiliations:** 1 Agenzia Nazionale per i Servizi Sanitari Regionali (AGENAS), Via Piemonte, 60, Rome, 00187 Italy; 2 Unicamillus International Medical University, Rome, Italy

**Keywords:** Person-centred care, Monitoring, Participatory evaluation, Citizens engagement, Quality improvement, Performance assessment

## Abstract

**Background:**

In 2018, a nationwide survey carried out in 387 acute care hospitals from 16 out of 21 Italian regions, allowed defining an extended checklist for the participatory evaluation of person-centredness in hospital care. We aimed to validate a reduced set of core items for continuous use across the country.

**Methods:**

Factor analysis was used to validate the construct of the checklist. Varimax rotation with eigenvalues > 1 was used to optimize factor structure. Items with an item-total correlation > 0.30 and factor loadings > 0.4 were attributed to individual factors. Items with inter-item correlation coefficient > 0.70 were submitted to expert opinion for final decision. Overall internal consistency was assessed through Cronbach’s alpha.

**Results:**

A total of 183 out of 243 items in the original checklist were submitted to factor analysis. A subgroup of 67 items was retained in 4 main areas, allocated as follows: 16 items in 4 sub-areas of “Person-oriented organizational and care processes”, 16 items in 4 sub-areas of “Physical accessibility, liveability and comfort of the facilities”, 15 items in 3 sub-areas of “Access to information, streamlining and transparency”, and 20 items in 4 sub-areas of “Taking care of the relationship with patients and citizens”. Overall values of Cronbach’s alpha ranged between 0.77 and 0.90, showing high consistency.

**Conclusions:**

This study validated a “core” checklist that can be routinely used to monitor the implementation of person-centred care in Italian hospitals. The tool can be applied more widely by multiple stakeholders as a measurement instrument for the participatory evaluation of person-centredness.

**Supplementary Information:**

The online version contains supplementary material available at 10.1186/s13690-024-01410-5.

**Table:** 

**Text box 1. Contributions to the literature**
• There are still variable definitions and a lack of practical instruments to measure the implementation of person-centred care in clinical settings for quality of care improvement.
• We have produced a checklist that has been successfully applied in acute care settings to evaluate the implementation of person-centredness with the active engagement of citizens, health professionals and policy makers.
• The contents of the checklist have been classified and optimised through item reduction techniques, delivering a tool that could be used to facilitate data collection and regular updating at national level.

## Background

Person-centredness, defined by the WHO as “*care which takes into account the preferences and aspirations of individual service users and the cultures of their communities*” [1], has become a fundamental dimension of quality of care targeted by national policies and international organizations (2–3).

Developments in this field show a growing interest towards its broad implementation in modern health systems. A recent review of the topic noted a progressive shift from conceptualisation to operationalization [4] confirming the trend towards person-centredness as a common driver of performance improvement [3].

In this context, the identification of indicators that are “fit for purpose”, paired with the availability of valid and reliable instruments to be used in the local context, are essential to monitor progress on a regular basis [5–8].

So far, the development of standardized approaches to reach this goal has been hampered by the lack of a unique definition [9, 10]. As a complex multidimensional construct, person-centredness encompasses multiple dimensions that have been differently defined and conceptualized over time [11–13] .

Consistently, separate instruments have been used to measure different aspect of care related to person centredness [4, 7]. In many cases, hospital care has been targeted as a key area of interest. Findings from recent reviews show that none of the surveys carried out in this context captured all eight dimensions of person-centred care identified by the Picker Institute [4, 14, 15]. Moreover, implementation appeared to be mostly limited at the clinical micro-level, where most often the professional culture, person-reported experiences (PREMs) and person-reported outcomes (PROMs) are adequately investigated and could be promptly applied [16, 17]. Consequently, there are very limited options available for decision makers to evaluate the overall implementation of person-centredness at both the the provider (meso) and governmental (macro) levels [4, 10].

In the decentralised context of the Italian National Health System (Sistema Nazionale Sanitario – SSN), the systematic evaluation of regional and national policies are highly relevant to ensure balanced governance [18]. In this context, the National Agency for Regional Health Services (AGENAS) adopted a holistic definition of person-centredness, intended as “*the commitment to make the provision of health services*,* including diagnostic and therapeutic programs*,* within settings of care that are as much as possible oriented towards the person*,* considered in the entirety of his/her physical*,* social and psychological dimensions*” [19] .

Recognising its relevance for new models of care delivery, AGENAS launched a national initiative to measure person-centredness in Italian hospitals, using a novel “checklist”, developed ad hoc to support a series of activities of participatory evaluation [19].

Multiple surveys coordinated by AGENAS in collaboration with Regions and Autonomous Provinces (R&AP) were carried to collect data in four different areas of interest: person-oriented organizational and care processes; physical accessibility, liveability, and comfort of facilities; access to information, streamlining and transparency; taking care of the relationship with patients and citizens [19–22].

The construction of the “checklist” was based on a consensus-driven approach, based on the opinion of experts, professionals and representatives of the citizens, who liaised with each other to incorporate the notions of “community knowledge” and “community ownership” into the final product [6, 7, 23–29].

The participatory process included the following steps to ensure face validity of the tool [19]:

- discussion to reach consensus on the initial composition of the checklist.
- pilot data collection using the checklist in 54 volunteering hospitals.
- revision of the checklist to identify a “long format” including 243 items.
- application of the checklist in 287 hospitals and evaluation of the results.

Following publication of the results [19], the stakeholders agreed that the “long format” of the checklist was not convenient for common use.

This study aims to identify a “core” version to facilitate broader adoption of the checklist, by responding to the following research questions:

- What is the factorial structure of the checklist according to construct validity?
- Which items should be included in a “core” checklist to facilitate the regular monitoring of person-centredness at a national scale?

The following sections present the results of the validation process.

## Methods

The present study used data collected in a national survey of person-centredness, carried out in years 2017–2018 in 387 accredited hospitals from 16 R&AP of Italy. used the data collected from the national survey of 2017–2018 because it provided the widest available data set to ensure the robustness of the statistical analyses. The study involved 841 professionals, 713 citizens, and 296 representatives of citizens and patient associations [19].

A tree-structured checklist was used to collect data from 243 items, classified in 4 main areas, 12 sub-areas, and 29 criteria. All items were formulated as questions with closed responses, mainly using binary options (presence-absence of a specific characteristic), except for few cases using multiple choices (three or more possible answers). A score between 0 and 10 was assigned to each item included in the checklist.

The checklist was used by a team of professionals and citizens specifically trained to conduct on-site visits for the participatory evaluation of facilities. The survey allowed assessing the presence/absence of the requirements included in the checklist, through the direct examination of documents collected in the occasion.

The present study used descriptive statistics of survey data e.g. the mean, standard deviation, median, range and coefficient of variation (expressed as a percentage to compare variation across different items).

The validation of the construct was based on the application of factor analysis in each area, to facilitate the interpretation of the results and reduction of the total number of items in the chechlist.

Any item not filled by at least 70% of responding hospitals (270 out of 387 participants in absolute terms) was excluded from factor analysis [30]. Those referred to specific departments/services e.g. paediatrics, obstetrics, oncology, intensive care, which were only available in specific hospitals, were considered “not applicable”.

Varimax rotation and eigenvalues greater than 1 were used to optimize the factorial structure [31]. A cut-off of 0.40 was used to attribute each item to a specific factor, reducing the likelihood of an overlap across multiple factors [27] .

In addition, we examined the homogeneity of each factor using the correlation of the item on the total, with the exclusion of items presenting a factor weight > 0.30.

Items with overlapping content and purpose were highlighted by an inter-item correlation coefficient greater or equal to 0. 70, indicating the need of choosing between items [32] .

Finally, the internal consistency of each area was examined using Cronbach’s Alpha, a coefficient measuring the homogeneity of the entire grouping of items [33] .

All statistical analyses were performed using the package “psych” of the R language [34] .

## Results

The characteristics of hospitals participating in the survey are described in Table 1.

**Table 1:** General characteristics of the study sample

Hospital Characteristic	Survey Sample of Hospitals		Total Population of Italian Hospitals*		
	*N*	%	*N*	%	
**N**	387	30.1	1,287	100.0	
**Macro Region**					
North	109	28.2	549	42.7	
Centre	98	25.3	259	20.1	
South, Islands	180	46.5	479	37.2	
**Type of Hospital**					
Public	251	64.9	595	46.2	
Trust	28	7.2	52	4.0	
Private	59	15.2	522	40.6	
Academic, Research	49	12.7	118	9.2	
**Volume (No.Beds)**					
Q1 (≤ 105)	98	25.3	665	51.7	
Q2 (> 105, ≤ 183)	97	25.1	283	22.0	
Q3 (> 183, ≤ 339)	95	24.5	229	17.8	
Q4 (> 339)	97	25.1	110	8.5	

* Italian Ministry of Health, Hospital Statistics, 1st January 2017 (http://www.dati.salute.gov.it/dati/dettaglioDataset.jsp?menu=dati&idPag=18)

A total of 387 facilities out of the 417 originally included in the survey were considered eligible for factor analysis. Among them, *N* = 251 (64.7%) were public hospitals, while *N* = 59 (15.2%) were private, *N* = 49 (12.7%) University Hospitals or Research Institutes (*N* = 49) and 28 (7.2%) non-university hospitals. Hospitals from the Centre and the South or from the Islands were more represented (25.3% and 46.5% respectively), compared to Northern Italy (28.2%).

Descriptive statistics for the complete checklist are shown in supplementary data (Tables A1.1-A1.4). The average item scores varied between 0.9 and 10, with a coefficient of variation ranging between 0 and 322.1 (item scores varied on average by more than 3 times the average for the same item).

For Area 1, the lowest average scores were recorded for “IT solution to share clinical data between facility and general practitioner” and “Multilingual panel/poster/notice for STP code for foreign users at Admission Office”. On average, higher values were reported for: “Religious assistance for Catholics” “Rooming-in available” and “Instruments for pain assessment available on medical record in Oncology”. Reduced variability was found for: “Religious assistance for Catholics”, “Instruments for pain assessment available on medical record in Oncology”. Greater variability was found for “Other areas dedicated to religious practice”.

In Area 2, the lowest average scores were measured for: “measures for access of blind/visually impaired to the Blood Tests and samples Service” and “Plan to remove sensory barriers”. On average, higher scores were found for: “ seats available in the waiting room of the Emergency Department”, access for disabled to the Imaging Service through at least 1pathway, “access for disabled to the Blood Tests and samples Service through at least one pathway”, “possibility to stop vehicle in front of main entry for those with walk limitations”. The minimum variability was recorded for: “seats available in the waiting room of the Emergency Department” ”, “Access for disabled to the Blood Tests and samples Service through at least one pathway”. The maximum variability was recorded for “Measures for access of blind/visually impaired to the Blood Tests and samples Service”.

In Area 3, the lowest average scores were recorded for: “Possibility to consult the personal medical record online and to download it”, pharmacies to pay services outside the healthcare facility” and “ATMs and/or other automatic cashiers to pay services outside the healthcare facility” “. On average, higher scores were found for: “Availability of digital images of radiological exams” and “drugs delivered to continue the prescribed therapy for visited or discharged patients”. The minimum variability was found for: “availability of digital images of radiological exams”,”drugs delivered to continue the prescribed therapy for visited or discharged patients”. The maximum variability was recorded for “possibility to consult the personal medical record online and to download it”.

Finally, for Area 4, the lowest average score was found for “Units where each patient is assigned to 1 + healthcare professionals during care”. Higher scores were observed for: “Presence of an operating procedure on informed consent” and “one or more education/information initiatives to promote breastfeeding among pregnant women”. The minimum variability was found for “one or more education/information initiatives to promote breastfeeding among pregnant women”, while the maximum variability was related to the “units where each patient is assigned to one or more healthcare professionals during care”.

A total of 48 items that were not filled by at least 70% (*N* = 270) of participating hospitals were excluded from factor analysis. Among them, 21 items were included in Area 1, 19 in Area 2, 1 in Area 3 and 7 in Area 4. Additional 13 items were also excluded for statistical reasons e.g. reduced variability (2 from area 1, 10 from area 2 and 1 in area 3).

At the end of all preliminary steps, a total of 183 items out of 243 originally included in the checklist were considered for factor analysis: 45 in Area 1, 57 in Area 2, 51 in Area 3 and 29 in Area 4.

The distribution of scores for all items included in the checklist is shown in supplementary data (Tables A2.1-A.2.4). The tables show the criteria used for the attribution of each item to a single factor, based on a grey scale used for the background of table cells. The attribution of an item to a specific factor (cell coloured in grey) has been based on the following conditions:

- Factor loading > 0.40.
- Standard deviation of the item > 0.30.

Factors not showing any value in a cell were excluded from factor analysis, due to insufficient variability e.g. all scores concentrated on 1 value.

For the final list of 183 items, Cronbach’s alpha showed good internal consistency [34] in each of the 4 areas, with values ranging between 0.77 for area 3 and 0.90 for area 1 (Table 2).

**Table 2:** Results of Cronbach Alpha by Reference Area

Area	Alpha (95% C.I.)
1	0.90 (0.88–0.91)
2	0.85 (0.83–0.87)
3	0.77 (0.74–0.80)
4	0.86 (0.84–0.88)

The results of factor analysis, including items renumbered according to the new structure, are presented in Table 3, 4, 5, 6.

**Table 3:** Validated checklist after item reduction: AREA 1 (* to be unified)

Area	Sub-Area	#	Item	Description
1. Person-oriented organizational and care processes	1.1 Relational support to multicultural needs	1	1.1.1*	Delivery of one or more training courses on pain management for clinicians during the last 36 months
		1	1.1.1*	Delivery of one or more training courses on pain management for nurses during the last 36 months
		2	1.1.2*	Availability of an interpreter to support translation (onsite or on call)
		3	1.1.3	Availability of documentation in multiple languages: one or more information sheets on available services (ED, obstetrician, etc.)
		4	1.1.4	Procedure for the provision of religious assistance to non-Catholics
		2	1.1.2*	Availability of services of cultural mediation (onsite or on call)
		5	1.1.5	Projects/activities to support the use of services offered by the care provider by users from other cultures
		6	1.1.6	Designation of people in charge of supporting the daily needs of patients using cross-border health care
	1.2 Supporting Materials	7	1.2.1*	Adoption of guidelines/procedures for pain management in General Medicine or stay at mid/low intensity of care
		7	1.2.1*	Adoption of guidelines/procedures for pain management in General Surgery or stay at mid intensity of care
		8	1.2.2	Adoption of guidelines/procedures for pain management in Emergency Department
		9	1.2.3	Information given to patients on post-surgery pain management
		10	1.2.4*	Possibility to choose special or customised menu on the basis of own ethical beliefs (e.g. vegetarian menu)
		10	1.2.4*	Possibility to choose special or customised menu on the basis of own religious beliefs (e.g. muslim menu)
	1.3 Privacy	11	1.3.1*	Bedrooms with visual separation (partial/total) between beds in General Medicine/medical stay at mid intensity of care
		11	1.3.1*	Bedrooms with visual separation (partial/total) between beds in General Surgery/surgery stay at mid intensity of care
	1.4 Access	12	1.4.1*	Visiting hour during weekdays
		12	1.4.1*	Visiting hour during weekends and holidays
		13	1.4.2	Availability of documents in multiple languages: one or more forms of informed consent
		14	1.4.3*	Availability of a multilingual panel/poster/notice at Reception/Information point/Patient and public relation and engagement Office, explaining how to obtain a special ID code (STP code) that would guarantee health care to foreign users not registered with the National Health Service and without a regular residence permit
		14	1.4.3*	Availability of a multilingual panel/poster/notice at Admission Office, explaining how to obtain a special ID code (STP code) to guarantee health care to foreign users not registered with the National Health Service and without a regular residence permit
		15	1.4.4	Presence of information leaflets delivered at one or more Reception points, explaining how to obtain a special ID code (STP code) that would guarantee health care to foreign users not registered with the National Health Service and without a regular residence permit
		16	1.4.5	Availability at the point of care of a printed notice written in multiple languages delivered to the citizen, indicating the office and procedure to obtain “STP code”

**Table 4:** Validated checklist after item reduction: AREA 2 (* to be unified)

Area	Sub-Area	#	Item	Description
2. Physical accessibility, liveability and comfort of the facilities	2.1 Services for the blind	17	2.1.1*	Presence of measures to aid blind and visually impaired accessing the Patient access centre
		17	2.1.1*	Presence of measures to aid blind and visually impaired accessing the Patient and public relation and engagement office
		17	2.1.1*	Presence of measures to aid blind and visually impaired accessing the Imaging Service
		17	2.1.1*	Presence of measures to aid visitors who are blind and visually impaired accessing the Blood test and samples Service
	2.2 Signposting at main hall	18	2.2.1	Presence in the entry hall of at least one updated signs indicating the location of the Patient and public relation and engagement
		19	2.2.2	Presence in the entry hall of at least one updated signs indicating the location of the imaging service
		20	2.2.3*	Presence in the entry hall of at least one updated signs showing the location of Gen.Medicine/medical stay at mid intensity of care
		20	2.2.3*	Presence in the entry hall of at least one updated signs showing the location of General Surgery/surgery stay at mid intensity of care
		21	2.2.4	Presence in the entry hall of at least one updated signs indicating the location of the Chief Medical Officer
	2.3 Comfort of indoor space	22	2.3.1*	Personal use of television in the room of General Medicine/medical stay at mid intensity of care
		22	2.3.1*	Personal use of television in the rooms of General Surgery/surgery stay at mid intensity of care
		23	2.3.2	Rooms with air conditioning in General Surgery/surgery stay at mid intensity of care
		24	2.3.3	Waiting room in emergency department with air conditioning
		25	2.3.4	Waiting room of blood tests and sample service with air conditioning
	2.4 Premium service	26	2.4.1	Taxi station close to the hospital, or automated system to call taxi available, or other tools to facilitate taxi calls
		27	2.4.2	Free internet access via wireless network
		28	2.4.3	Posting of timetable of toilet daily cleaning with name/ID operator and time at the Patient Access Centre
		29	2.4.4	Possibility for patients and family to buy newspapers and magazines
		30	2.4.5	Possibility for patients and family to buy essential accessories for personal care
		31	2.4.6	Presence of a coffee bar
		32	2.4.7	Presence of library/reading area with books and magazines freely accessible for patients and family

**Table 5:** Validated checklist after item reduction: AREA 3 (* to be unified)

Area	Sub-Area	#	Item	Description
3. Access to information, streamlining and transparency	3.1 Direct use	33	3.1.1	Presence of alternative modes of payment for services available at the facility: automated cash machines at the facility
		34	3.1.2	Possibility to receive laboratory test results at home
		35	3.1.3	Possibility to receive laboratory test results online
		36	3.1.4	Possibility to receive the personal medical record at home
		37	3.1.5	Patient and public relation and engagement Office on a continued scheduled one or more times per week
		38	3.1.6	Presence at the hospital website of a list of online services available
		39	3.1.7	Presence at the hospital website of a list of documents required to request a copy of the personal medical record
		40	3.1.8	Presence at the hospital website of forms to request a copy of the personal medical record (download)
		41	3.1.9	Presence at the hospital website of alternatives equivalent to audio/visual content
	3.2 Extended time for the provision of services	42	3.2.1	Possibility to book health services at the facility through one or more office counter of the Patient Access Centre open more than 36 h per week
		43	3.2.2	Possibility to book health services at the facility through one or more office counter of the Patient Access Centre on a continued schedule one or more times per week
		44	3.2.3	Possibility to book health services at the facility through one or more office counter of the Patient Access Centre open before 9AM one or more times per week
		45	3.2.4	Patient and public relation and engagement Office open more than 36 h per week
	3.3 Extended territorial coverage of booking services	46	3.3.1	Possibility for users to book services provided in hospital at Patient Access centre located in Local Health Facility
		47	3.3.2	Possibility for users to book services provided in hospital at Patient Access Centre via pharmacy

**Table 6:** Validated checklist after item reduction: AREA 4 (* to be unified)

Area	Sub-Area	#	Item	Description
4. Taking care of the relationship with patients and citizens	4.1 Satisfaction surveys	48	4.1.1	Delivery of a satisfaction survey of users of the facility during the last 24 months
		49	4.1.2	Public disclosure of results of the satisfaction survey of facility users
		50	4.1.3	Implementation of one or more actions for improvement following the survey
	4.2 Training on relation and empowerment	51	4.2.1	Delivery of one or more initiatives of health promotion promoted in collaboration with civic organizations
		52	4.2.2	Delivery of one or more initiatives formally presented during the last 24 months in the scientific/institutional/civic contexts as promoted by the hospital to encourage patient empowerment
		53	4.2.3	Delivery of one or more training courses during the last 36 months on cultural diversity for health professionals dealing with foreign patients
		54	4.2.4	Delivery of one or more training courses during the last 36 months on clinical communication and/or helping relationship for clinicians
		55	4.2.5	Delivery of one or more training courses during the last 36 months on clinical communication and/or helping relationship for nurses
		56	4.2.6	Delivery of one or more training courses during the last 36 months for front office personnel on relation and communication with users
	4.3 Service Charter	57	4.3.1	Availability of the Service Charter at the facility
		58	4.3.2	Availability of the Service Charter at the official website
		59	4.3.3	Presence of a Service Charter updated maximum 36 months ago
		60	4.3.4	Presence of a Service Charter including general information on the services provided and procedures to access and use services
		61	4.3.5	Presence of a Service Charter including a section on commitments and related indicators, standard and evaluation tools
		62	4.3.6	Presence of a Service Charter with a section dedicated to safeguarding the citizen/user from inefficiencies/acts/behaviours that may limit the fruition of services
	4.4 Stewardship	63	4.4.1	Presence of regular reports to monitor the application of the trust operating procedures on informed consent
		64	4.4.2	Units with a defined welcome procedure
		65	4.4.3	Units/wards where one or more reference health professionals (clinician/nurse) are assigned to each patient during care
		66	4.4.4	Presence of an “Info Point” in the main entry hall
		67	4.4.5	Presence of a welcome procedure

The “core” checklist includes a total of 67 items, classified in 4 main areas and 15 sub-areas, named as follows:

- Area 1, “Person-oriented organizational and care processes " including 16 items in 4 sub-areas:
  - 1.1 “Relational supports to multicultural needs” (6 items).
  - 1.2 “Supporting materials” (4 item).
  - 1.3 “Privacy " (1 item).
  - 1.4 “Access” (5 item).
- Area 2, “Physical accessibility, liveability and comfort of the facilities” including 16 items in 4 sub-areas:
  - 2.1 “Services for the blind” (1 item).
  - 2.2 “Signposting at main hall " (4 items).
  - 2.3 “Comfort of indoor space” (4 items).
  - 2.4 “Premium Service” (7 items).
- Area 3, “Access to information, simplification and transparency” including 15 items in 3 sub-areas:
  - 3.1 “Direct use” (9 items).
  - 3.2 “Extended time for the provision of services” (4 items).
  - 3.3 “Extended territorial coverage of booking services” (2 items).
- Area 4, “Taking care of the relationship with patients and citizens” including 20 items in 4 sub-areas:
  - 4.1 “Satisfaction surveys” (83 items).
  - 4.2 “Training on relation and empowerment” (6 items).
  - 4.3 “Service Charter“(6 items).
  - 4.4 “Stewardship " (5 items).

## Discussion

The results emerging from the validation of the checklist performed for this study confirm the relevance of organizational aspects for the implementation of person-centredness [19].

However, adopting a tool that should cover all aspects of a “holistic” definition of person-centredness may prove to be difficult, due to the high number of items involved. In the case of our checklist, the specification of 243 items for each hospital, required a substantial organizational effort by providers, professionals, and citizens, who jointly committed to all phases of co-design of improvement plans.

This study responds to the practical need of repeating the survey at regular intervals across the whole national territory.

Regarding the first research question, the application of factor analysis confirmed the overall structure of the “long format” previously specified, including the four main areas originally outlined: (1) person-oriented organizational and care processes; (2) Physical accessibility, liveability and comfort of the facilities; (3) Access to information, streamlining and transparency; and (4) Taking care of the relationship with patients and citizens.

As far as the second research question is concerned, the reduction of the checklist to 67 core items identified an agile instrument that could be used for participatory evaluation more easily.

Practical implications of items reduction include the possibility to apply a simpler data sheet at hospital level, without losing the capacity to cover all main dimensions of person-centredness. By applying the proposed protocol of participatory assessment, citizens and professionals may arrange a one-day visit at hospitals, to fill the checklist through the direct observation of wards and reading the documentation available on site.

A reduced number of items may simplify the process, by optimising the time and resources spent by professionals and citizens. A more sustainable effort may encourage public participation, while increasing the potential number of hospitals involved in the evaluation, to provide better coverage at national level.

In this context, key measures can capture complex phenomena effectively, by highlighting critical issues that should be prioritised, as opposed to specific aspects that need more accurate examination through an expanded list of items.

For example, a facility showing shortcomings with regards to multicultural communication, can envisage an expansion of data collection to cover all items included in the original checklist.

The evaluation of person-centredness in its broader conceptualization can help the subsequent specification of improvement plans. To this end, two different versions of the checklist are available, which can be conveniently used according to the needs of different evaluation teams.

The application of the core list is practically convenient, as it includes only 15–20 items for each section, highlighting the key improvements needed to improve services “that matters to the person”.

The “core” checklist and all associated materials are publicly available at a dedicated page of the main AGENAS website (https://www.agenas.gov.it/aree-tematiche/qualita-e-sicurezza/empowerment-del-cittadino/valutazione-partecipata-2022-2023).

The availability of the tool facilitates its uptake by R&AP, in preparation of new plans for the implementation of person-centred health care at national level. In this framework, AGENAS will take stock of its experience to transfer knowledge among all interested parties, by offering tools that can facilitate the data collection of essential items for comparative analysis at different levels [35].

The standardization process induced by AGENAS in complex areas of performance indicators e.g. patient safety has already shown how R$AP need to collaborate and share experiences to improve the consistency of all results [36, 37].

The continuous monitoring of person-centredness in Italian hospitals is particularly useful in the phase of recovery from the emergency of the COVID19 pandemic, ensuring that health care providers are aligned with the expectations of patients, in the transition towards person-centred health systems [2, 8, 38, 39].

Finally, a clarification is needed with regards to the methodological aspects related to our application of factor analysis for item reduction vs. exploratory/confirmatory approaches.

The proposed instrument is not aimed at gathering opinions from individuals, but represents a tool for sustainable data collection of structural characteristics at hospital level. The checklist measures aspects that are almost entirely objective e.g. services that do/do not exist in a hospital. Therefore, psychometric standards involving random variability and/or interrater agreement do not generally apply [40]. On the other hand, what is relevant in this specific context, is using the correlation structure as an item reduction technique, allowing to discard redundant items to generate a new tool that can aid both measurement and interpretation in the evaluation of person-centred services.

Finally, some limitations of the proposed approach are worth to be outlined.

Firstly, the validated checklist may need regular updates over time, to avoid considering it as a permanent tool for long term data collection. Nevertheless, its validation was based on a large national sample that can be considered reliable for use in the short/mid-term, for those aspects covered by the checklist.

Secondly, the requirement of including only items filled by at least 70% hospitals has involved the elimination of 47 items that could have been used to evaluate specific facilities e.g. specialised centres of paediatrics, obstetrics, oncology and intensive care. More studies are needed to consider these types of services as “supplementary modules” of the checklist.

Thirdly, the final version of the checklist required specific decisions regarding items that were strongly correlated with each other (inter-item correlation equal or above 0.70). Following the statistical validation, items falling in this category (see Table 3.1) were submitted for further examination to field experts. Briefly, we applied a qualitative approach by asking directly to a pool of national experts which item, between those highly correlated in each sub-area, should be kept in the checklist. Experts were selected on the basis of their direct experience in participatory assessment and coordination activities at national and regional level. A total of five experts participated to the consultation process. Items with the highest number of preferences were retained in the final version of the checklist.

## Conclusions

This study validated a “core” checklist for the participatory assessment of person-centred hospital care, highlighting a manageable number of items applicable on a regular basis for many hospitals.

The national experience of Italy can provide valuable lessons regarding the applicability of participatory approaches at national level, offering a validated tool that can be used in other contexts.

The checklist can be immediately used by Regions and Autonomous Provinces for next rounds of participatory evaluation of person-centred care.

## Electronic supplementary material

Below is the link to the electronic supplementary material.

Supplementary Material 1

## Data Availability

No datasets were generated or analysed during the current study.

## Abbreviations

AGENAS: National Agency for Regional Health Services
IT: Information technology
STP: Foreigners temporarily present (“Straniero Temporaneamente Presente”)
ATM: Automated Teller Machine
