# Supplementary material for: A framework for the continuous monitoring of person-centred hospital care: validation of a checklist for participatory service improvement
Source: Arch Public Health. 2025 Jan 14;83:12. doi: 10.1186/s13690-024-01410-5 (PMC11730461; doi:10.1186/s13690-024-01410-5)
Supplement: Supplementary file 1 — Supplementary Material 1 [file 13690_2024_1410_MOESM1_ESM.docx]

**Table A1.1- Descriptive statistics - items AREA 1 (sd: standard deviation; CV: coefficient of variation x 100)**

| **Description** | **Code** | **Mean** | **Min** | **Max** | **sd** | **CV** |
| --- | --- | --- | --- | --- | --- | --- |
| Psychological support for cancer patients | 1.1.1.1 | 7.0 | 0 | 10 | 3.2 | 456 |
| Psychological support for victims of sexual violence | 1.1.1.2 | 7.9 | 0 | 10 | 3.3 | 41 |
| Psychological support for inpatients waiting/receiving organ transplant | 1.1.1.3 | 6.7 | 0 | 10 | 2.9 | 43 |
| Psychological support for children/adolescents in ordinary hospitalization | 1.1.1.4.1 | 6.6 | 0 | 10 | 3.1 | 48 |
| Psychological support for children/adolescents in Day hospital/Day Surgery | 1.1.1.4.2 | 6.4 | 0 | 10 | 3.2 | 50 |
| Psychological support for pregnancy-related problems | 1.1.1.5 | 7.0 | 0 | 10 | 3.2 | 46 |
| Projects to connect the hospital to the outside world | 1.1.2.6 | 5.6 | 0 | 10 | 4.3 | 76 |
| Projects to ensure educational needs/continuity at school | 1.1.2.7 | 6.6 | 0 | 10 | 4.7 | 71 |
| Projects to support playing and positive emotions in children | 1.1.2.8 | 8.5 | 0 | 10 | 3.0 | 35 |
| Visiting hours during weekdays | 1.1.2.9.1 | 4.2 | 0 | 10 | 4.0 | 93 |
| Visiting hours during weekends and holidays | 1.1.2.9.2 | 4.8 | 0 | 10 | 4.0 | 82 |
| Pregnant women can be supported by a trusted person in the labor room | 1.1.3.10.1 | 9.3 | 0 | 10 | 2.6 | 28 |
| Pregnant women can be supported by a trusted person in delivery room | 1.1.3.10.2 | 9.6 | 0 | 10 | 2.1 | 21 |
| Rooming-in available | 1.1.3.11 | 9.7 | 0 | 10 | 1.7 | 18 |
| Parent can assist own child in NICU without time limitation | 1.1.3.12 | 6.5 | 0 | 10 | 4.8 | 75 |
| Parent can assist own admitted child without time limitation | 1.1.3.13 | 9.0 | 0 | 10 | 2.1 | 23 |
| Parent can stay in pre-anaesthesia room/recovery room for invasive tests | 1.1.3.14.1 | 7.6 | 0 | 10 | 4.3 | 57 |
| Parent can stay in recovery room for invasive tests/surgery | 1.1.3.14.2 | 7.4 | 0 | 10 | 4.4 | 60 |
| Procedures on supplemental non-medical care | 1.1.3.15 | 7.8 | 0 | 10 | 4.1 | 53 |
| Open-Intensive-Care-Unit model is applied by extending visiting hours | 1.1.3.16 | 4.0 | 0 | 10 | 3.2 | 81 |
| Open-Intensive-Care-Unit model is applied by eliminating barrier devices | 1.1.3.17 | 5.2 | 0 | 10 | 5.0 | 97 |
| Agreements with accommodation facilities to offer reduced prices | 1.1.3.18 | 4.4 | 0 | 10 | 5.0 | 113 |
| Instruments for pain assessment available on medical record in General Medicine | 1.1.4.19.1 | 9.6 | 0 | 10 | 2.0 | 21 |
| Instruments for pain assessment available on medical record in General Surgery | 1.1.4.19.2 | 9.6 | 0 | 10 | 2.0 | 21 |
| Instruments for pain assessment available on medical record in Oncology | 1.1.4.19.3 | 9.7 | 0 | 10 | 1.7 | 17 |
| Guidelines/procedures for pain management adopted in General Medicine | 1.1.4.20.1 | 8.5 | 0 | 10 | 3.5 | 41 |
| Guidelines/procedures for pain management adopted in General Surgery | 1.1.4.20.2 | 8.8 | 0 | 10 | 3.2 | 36 |
| Guidelines/procedures for pain management adopted in Oncology | 1.1.4.20.3 | 9.0 | 0 | 10 | 3.0 | 33 |
| Guidelines/procedures for pain management adopted in Emergency Room | 1.1.4.20.4 | 8.3 | 0 | 10 | 3.7 | 45 |
| Labour analgesia guaranteed 24 hours a day with an anaesthesiologist | 1.1.4.21 | 6.2 | 0 | 10 | 4.5 | 74 |
| 1+ training courses on pain management for doctors last 36 months | 1.1.4.22.1 | 8.3 | 0 | 10 | 3.8 | 46 |
| 1+ training courses on pain management for nurses last 36 months | 1.1.4.22.2 | 8.3 | 0 | 10 | 3.7 | 45 |
| Information on post-surgery pain management | 1.1.4.23 | 8.2 | 0 | 10 | 3.8 | 46 |
| Activities to inform women about their right to give birth anonymously | 1.2.1.24 | 9.1 | 0 | 10 | 2.8 | 31 |
| Materials to communicate people accessing the ER they can nominate who can be informed | 1.2.1.25 | 7.9 | 0 | 10 | 4.1 | 51 |
| Materials to communicate people admitted to inpatient stay they can nominate who can be informed | 1.2.1.26 | 9.0 | 0 | 10 | 3.0 | 33 |
| Actions to prevent calling by name patients waiting at the blood tests and samples service from being called by name | 1.2.1.27 | 9.6 | 0 | 10 | 2.0 | 21 |
| Measures to limit the visibility of patients in ICU during visiting hours | 1.2.2.28 | 9.6 | 0 | 10 | 2.0 | 21 |
| Rooms reserved for terminal patients and family in General Medicine | 1.2.2.29 | 5.6 | 0 | 10 | 5.0 | 90 |
| Visual separation (partial or total) between beds in General Medicine | 1.2.2.30.1 | 5.9 | 0 | 10 | 4.2 | 71 |
| Visual separation (partial or total) between beds in General Surgery | 1.2.2.30.2 | 5.9 | 0 | 10 | 4.4 | 74 |
| Visual separation (partial or total) between beds in (specify) | 1.2.2.30.3 | 6.5 | 0 | 10 | 4.3 | 66 |
| Availability of an interpreter (onsite or on call) | 1.3.1.31 | 7.7 | 0 | 10 | 4.2 | 55 |
| 1+ forms of informed consent available in different languages | 1.3.1.32.1 | 4.3 | 0 | 10 | 4.9 | 116 |
| 1+ information sheets on available services (ED, obstetrics) | 1.3.1.32.2 | 5.6 | 0 | 10 | 5.0 | 89 |
| Service charter available in different languages | 1.3.1.32.3 | 4.1 | 0 | 10 | 4.9 | 121 |
| Discharge letter released in foreign language to requesting cross-border patients | 1.3.1.33 | 3.8 | 0 | 10 | 4.3 | 115 |
| Religious assistance for Catholics | 1.3.2.34 | 10.0 | 0 | 10 | 0.7 | 7 |
| Catholic Chapel available | 1.3.2.35.1 | 9.6 | 0 | 10 | 1.9 | 20 |
| Other areas dedicated to religious practice | 1.3.2.35.2 | 2.2 | 0 | 10 | 4.2 | 186 |
| Procedure for religious assistance for non-Catholics | 1.3.2.36 | 4.5 | 0 | 10 | 5.0 | 111 |
| Possibility to perform funerary functions according to ethical beliefs or faith (including body reconstruction by relatives). | 1.3.2.37 | 7.6 | 0 | 10 | 4.3 | 56 |
| Availability of services of cultural mediation (onsite or on call) | 1.3.3.38 | 6.9 | 0 | 10 | 4.6 | 66 |
| Projects to support use of services by users from other cultures | 1.3.3.39 | 4.0 | 0 | 10 | 4.6 | 114 |
| Possibility to choose or customise menu based on ethical preferences | 1.3.3.40.1 | 9.1 | 0 | 10 | 2.8 | 31 |
| Possibility to choose or customise menu based on religious preferences | 1.3.3.40.2 | 8.8 | 0 | 10 | 3.3 | 37 |
| Multilingual panel/poster/notice for STP code for foreign users (STP code) at Reception/information point/Patient and public relation and engagement Office | 1.3.3.41.1 | 4.1 | 0 | 10 | 4.9 | 121 |
| Multilingual panel/poster/notice for STP code for foreign users at Admission Office | 1.3.3.41.2 | 3.4 | 0 | 10 | 4.7 | 141 |
| Information leaflets on STP code for foreign users at 1+ Receptions | 1.3.3.41.3 | 3.7 | 0 | 10 | 4.8 | 130 |
| Website explaining how to obtain an STP code for foreign users | 1.3.3.41.4 | 5.4 | 0 | 10 | 5.0 | 92 |
| Multilingual printed notice at Point of Care indicating office and procedure for STP code | 1.3.3.41.5 | 3.4 | 0 | 10 | 4.8 | 139 |
| Designation of people supporting daily needs of patients using cross-border health care | 1.3.3.42 | 3.9 | 0 | 10 | 4.9 | 124 |
| Day Surgery discharge summary with details on who to refer to if needed | 1.4.1.43 | 9.6 | 0 | 10 | 1.9 | 20 |
| 1+ procedures to ensure continuity in the transition from another setting | 1.4.1.44 | 9.7 | 0 | 10 | 1.8 | 19 |
| Periodic audit reports on discharge with transition from another setting | 1.4.1.45 | 6.8 | 0 | 10 | 4.68 | 69 |
| Possibility to book post-discharge services through hospital doctor | 1.4.1.46 | 9.1 | 0 | 10 | 2.88 | 32 |
| Periodic reports on booking of post-hospitalization visits/exams | 1.4.1.47 | 4.5 | 0 | 10 | 4.98 | 111 |
| IT solution to share clinical data between facility and general practitioner | 1.4.1.48 | 2.4 | 0 | 10 | 4.29 | 177 |

**Table A1.2- Descriptive statistics - items AREA 2 (sd: standard deviation; CV: coefficient of variation x 100)**

| **Description** | **Code** | **Mean** | **Min** | **Max** | **sd** | **CV** |
| --- | --- | --- | --- | --- | --- | --- |
| Access for disabled to the Patient access centre through at least 1 pathway | 2.1.1.49.1 | 9.6 | 0 | 10 | 1.9 | 19 |
| Access for disabled to the Patient and public relation and engagement Office through at least 1 pathway | 2.1.1.49.2 | 9.6 | 0 | 10 | 2.0 | 21 |
| Access for disabled to the Imaging Service through at least 1pathway | 2.1.1.49.3 | 9.9 | 0 | 10 | 1.0 | 10 |
| Access for disabled to the Blood Tests and samples Service through at least 1pathways | 2.1.1.49.4 | 9.9 | 0 | 10 | 0.8 | 8 |
| Measures for access of blind/visually impaired to the Patient Access Centre | 2.1.1.50.1 | 1.7 | 0 | 10 | 3.8 | 219 |
| Measures for access of blind/visually impaired to the Patient and public relation and engagement Office | 2.1.1.50.2 | 1.7 | 0 | 10 | 3.8 | 220 |
| Measures for access of blind/visually impaired to the Imaging Service | 2.1.1.50.3 | 1.7 | 0 | 10 | 3.8 | 221 |
| Measures for access of blind/visually impaired to the Blood Tests and samples Service | 2.1.1.50.4 | 1.3 | 0 | 10 | 3.4 | 255 |
| Plan to remove architectural barriers | 2.1.1.51.1 | 5.1 | 0 | 10 | 5.0 | 99 |
| Plan to remove sensory barriers | 2.1.1.51.2 | 1.6 | 0 | 10 | 3.2 | 198 |
| Parking available for people accessing the facility | 2.1.2.52 | 7.3 | 0 | 10 | 3.0 | 41 |
| Area for cycles and/or motorcycles parking | 2.1.2.53 | 8.7 | 0 | 10 | 3.3 | 38 |
| Reserved parking slots for disabled people accessing the facility | 2.1.2.54 | 9.7 | 0 | 10 | 1.3 | 13 |
| Reserved parking slots for pregnant women ("pink parking") | 2.1.2.55 | 6.1 | 0 | 10 | 4.7 | 77 |
| 1 + public transport lines available to get to the hospital | 2.1.2.56 | 9.5 | 0 | 10 | 2.2 | 23 |
| Free bus shuttle service for users and visitors to move between pavilions of the facility | 2.1.2.57 | 3.6 | 0 | 10 | 4.8 | 134 |
| Taxi station close to the hospital, systems to call taxi, other tools | 2.1.2.58 | 7.0 | 0 | 10 | 4.6 | 65 |
| Possibility to stop vehicle in front of main entry for those with walk limitations | 2.1.2.59 | 9.9 | 0 | 10 | 1.0 | 10 |
| Internal pedestrian pathway or sidewalk | 2.1.2.60 | 9.4 | 0 | 10 | 2.4 | 26 |
| Main hall with 1+ updated signs indicating the location of the Patient Access Centre | 2.2.1.61.1 | 9.4 | 0 | 10 | 2.4 | 26 |
| Main hall with 1+ signs indicating the location of t the Patient and public relation and engagement Office | 2.2.1.61.2 | 8.9 | 0 | 10 | 3.2 | 36 |
| Main hall with 1+ signs indicating the location of the Imaging Service | 2.2.1.61.3 | 9.5 | 0 | 10 | 2.1 | 22 |
| Main hall with 1+ signs indicating the location of the General Medicine Unit | 2.2.1.61.4 | 9.4 | 0 | 10 | 2.4 | 26 |
| Main hall with 1+ signs indicating the location of the General Surgery Unit | 2.2.1.61.5 | 9.5 | 0 | 10 | 2.2 | 24 |
| Main hall with 1+ signs indicating the location of the General Direction | 2.2.1.61.6 | 9.1 | 0 | 10 | 2.8 | 31 |
| Internal signs showing pathways for different destination areas | 2.2.1.62 | 7.5 | 0 | 10 | 4.3 | 58 |
| Internal pathways allowing inpatients of General Surgery Unit to reach operating room | 2.2.2.63.1 | 9.4 | 0 | 10 | 2.4 | 25 |
| Internal pathways allowing pregnant women to reach the delivery/operating room | 2.2.2.63.2 | 9.7 | 0 | 10 | 1.7 | 18 |
| Internal pathways allowing inpatients to reach the Imaging Service (without passing through the waiting room) | 2.2.2.64 | 7.0 | 0 | 10 | 4.6 | 66 |
| Personal use of television in rooms of General Medicine | 2.3.1.65.1 | 3.0 | 0 | 10 | 4.3 | 143 |
| Personal use of television in rooms of General Surgery | 2.3.1.65.2 | 3.5 | 0 | 10 | 4.6 | 132 |
| Personal use of television in rooms of the Unit (specify) ………… | 2.3.1.65.3 | 4.3 | 0 | 10 | 4.7 | 109 |
| Rooms with air conditioning system in General Medicine | 2.3.1.66.1 | 8.4 | 0 | 10 | 3.5 | 42 |
| Rooms with air conditioning in General Surgery | 2.3.1.66.2 | 8.6 | 0 | 10 | 3.4 | 39 |
| Rooms with air conditioning in the Unit (specify)………… | 2.3.1.66.3 | 9.1 | 0 | 10 | 2.8 | 30 |
| Rooms with no more than 2 beds in the General Medicine Unit | 2.3.1.67.1 | 6.3 | 0 | 10 | 3.4 | 55 |
| Rooms with no more than 2 beds in the General Surgery Unit | 2.3.1.67.2 | 6.5 | 0 | 10 | 3.5 | 53 |
| Rooms with no more than 2 beds in the Unit (specify)………… | 2.3.1.67.3 | 7.1 | 0 | 10 | 3.6 | 50 |
| Free internet access via wireless network | 2.3.1.68 | 4.4 | 0 | 10 | 5.0 | 114 |
| Rooms with internal bathrooms in the General Medicine Unit | 2.3.1.69.1 | 8.4 | 0 | 10 | 3.2 | 39 |
| Rooms with internal bathrooms in the General Surgery Unit | 2.3.1.69.2 | 8.6 | 0 | 10 | 3.1 | 36 |
| Rooms with internal bathrooms in the Unit (specify)………… | 2.3.1.69.3 | 8.7 | 0 | 10 | 2.9 | 34 |
| Dining room reserved for patients and their families in the Psychiatric Unit | 2.3.1.70 | 8.7 | 0 | 10 | 3.4 | 39 |
| Sitting room or any other area for social activities in the Psychiatric Unit | 2.3.1.71 | 8.6 | 0 | 10 | 3.5 | 40 |
| Paediatric Units equipped with play area | 2.3.2.72.1 | 9.2 | 0 | 10 | 2.6 | 28 |
| Paediatric Units equipped with reading corners/ library | 2.3.2.72.2 | 8.4 | 0 | 10 | 3.5 | 42 |
| Paediatric Units equipped with furniture suitable for children | 2.3.2.73.1 | 9.3 | 0 | 10 | 2.5 | 27 |
| Paediatric Units equipped with coloured walls and corridors | 2.3.2.73.2 | 9.6 | 0 | 10 | 2.0 | 21 |
| Paediatric Units equipped with specific decorations on the walls | 2.3.2.73.3 | 9.5 | 0 | 10 | 2.1 | 22 |
| Waiting room dedicated for children in the Emergency room | 2.3.2.74 | 6.4 | 0 | 10 | 4.8 | 75 |
| Child-friendly operating room | 2.3.2.75 | 4.5 | 0 | 10 | 5.0 | 112 |
| Possibility for patients on a free diet to choose 2+ menu options | 2.3.3.76 | 9.7 | 0 | 10 | 1.7 | 17 |
| Breakfast time | 2.3.3.77 | 9.8 | 0 | 10 | 1.2 | 13 |
| Lunch time | 2.3.3.78 | 8.9 | 0 | 10 | 3.1 | 34 |
| Dinner time | 2.3.3.79 | 2.7 | 0 | 10 | 4.5 | 163 |
| Obtaining extra cleaning services on demand by patient and/or health professionals | 2.3.3.80 | 9.7 | 0 | 10 | 1.6 | 16 |
| Staff name/code and timing of daily bathroom cleaning shown in Emergency Room | 2.3.3.81.1 | 4.6 | 0 | 10 | 5.0 | 108 |
| Staff name/code and timing of daily bathroom cleaning shown in Patient Access Centre | 2.3.3.81.2 | 4.5 | 0 | 10 | 5.0 | 110 |
| Patients can receive meal and/or food during visit in Emergency Room | 2.3.3.82.1 | 5.9 | 0 | 10 | 4.9 | 83 |
| Patients can receive meal and/or food during visit in Outpatient Clinic | 2.3.3.82.2 | 8.7 | 0 | 10 | 3.3 | 38 |
| Inpatients and their family can buy newspapers and magazines | 2.4.1.83 | 6.5 | 0 | 10 | 4.8 | 73 |
| Inpatients and their family can buy essential accessories for personal care | 2.4.1.84 | 6.2 | 0 | 10 | 4.8 | 78 |
| ATM inside the facility | 2.4.1.85 | 5.2 | 0 | 10 | 5.0 | 96 |
| Safety deposit service for inpatients | 2.4.1.86 | 4.2 | 0 | 10 | 4.3 | 102 |
| Barber/hairdresser available inside facility or on call | 2.4.1.87 | 4.4 | 0 | 10 | 4.6 | 104 |
| Coffee bar | 2.4.1.88 | 7.1 | 0 | 10 | 4.5 | 64 |
| Reading corner with books/magazines freely available to inpatients/family | 2.4.1.89 | 4.2 | 0 | 10 | 4.3 | 103 |
| Green area / garden with benches accessible to inpatients and family | 2.4.1.90 | 6.9 | 0 | 10 | 4.5 | 65 |
| Adequate dedicated space in the Waiting room of the Emergency Department | 2.4.2.91.1 | 9.6 | 0 | 10 | 1.9 | 20 |
| Seats available in the waiting room of the Emergency Department | 2.4.2.91.2 | 10.0 | 10 | 10 | 0.0 | 0 |
| Food vending machines available in the Waiting room of Emergency Department | 2.4.2.91.3 | 9.1 | 0 | 10 | 2.8 | 31 |
| Air conditioning available in the waiting room of Emergency Department | 2.4.2.91.4 | 9.3 | 0 | 10 | 2.6 | 28 |
| Display counter available in the waiting room of Emergency Department with real time number of patients waiting | 2.4.2.91.5 | 7.0 | 0 | 10 | 4.6 | 66 |
| Television available in the waiting room of Emergency Department | 2.4.2.91.6 | 5.8 | 0 | 10 | 5.0 | 86 |
| Adequate dedicated space in the Waiting room of Blood Tests and samples Service | 2.4.2.92.1 | 8.8 | 0 | 10 | 3.2 | 36 |
| Seats available in the waiting room of Blood Tests and samples Service | 2.4.2.92.2 | 9.8 | 0 | 10 | 1.4 | 15 |
| Food vending machines available in the Waiting room of Blood Tests and samples Service | 2.4.2.92.3 | 8.2 | 0 | 10 | 3.9 | 47 |
| Air conditioning available in the waiting room of Blood Test and samples Service | 2.4.2.92.4 | 8.7 | 0 | 10 | 3.3 | 38 |
| Adequate dedicated space in the Waiting room of Intensive Care | 2.4.2.93.1 | 7.7 | 0 | 10 | 4.2 | 55 |
| Seats available in the waiting room of Intensive Care | 2.4.2.93.2 | 9.3 | 0 | 10 | 2.6 | 28 |
| Food vending machines available in the Waiting room of Intensive Care | 2.4.2.93.3 | 7.0 | 0 | 10 | 4.6 | 66 |
| Air conditioning available in the waiting room of Intensive Care | 2.4.2.93.4 | 8.4 | 0 | 10 | 3.7 | 44 |
| Adequate dedicated space in the Waiting room of Delivery Room | 2.4.2.94.1 | 6.9 | 0 | 10 | 4.6 | 67 |
| Seats available in the waiting room of Delivery Room | 2.4.2.94.2 | 9.0 | 0 | 10 | 3.0 | 34 |
| Food vending machines available in the Waiting room of Delivery Room | 2.4.2.94.3 | 6.7 | 0 | 10 | 4.7 | 70 |
| Air conditioning available in the waiting room of Delivery Room | 2.4.2.94.4 | 8.0 | 0 | 10 | 4.0 | 51 |

**Table A1.3- Descriptive statistics - items AREA 3 (sd standard deviation; CV: coefficient of variation x 100)**

| **Description** | **Code** | **Mean** | **Min** | **Max** | **d.s.** | **CV** |
| --- | --- | --- | --- | --- | --- | --- |
| Health services booked at 1+ office counter of Patient Access Centre open >36hrs per week | 3.1.1.95.1 | 7.5 | 0 | 10 | 4.3 | 58 |
| Health services booked at 1+ office counter of Patient Access Centre open all day 1+ days per week | 3.1.1.95.2 | 7.0 | 0 | 10 | 4.6 | 66 |
| Health services booked at 1+ desks of Patient Access Centre open on Saturdays | 3.1.1.95.3 | 6.3 | 0 | 10 | 4.8 | 77 |
| Health services booked at 1+ office counter of Patient Access Centre before 9AM 1+ days per week | 3.1.1.95.4 | 9.3 | 0 | 10 | 2.5 | 26 |
| Health services booked at 1+ office counter of Patient Access Centre closing 5PM 1+ days per week | 3.1.1.95.5 | 6.6 | 0 | 10 | 4.8 | 73 |
| Services booked at Patient Access Centre at a office counter with dedicated operator | 3.1.1.96.1 | 8.9 | 0 | 10 | 3.1 | 35 |
| Services booked at the Patient Access Centre by phone | 3.1.1.96.2 | 9.5 | 0 | 10 | 2.2 | 23 |
| Services booked at Patient Access Centre through the General Practitioner | 3.1.1.96.3 | 3.2 | 0 | 10 | 4.7 | 145 |
| Services booked at Patient Access Centre through a Pharmacy | 3.1.1.96.4 | 4.7 | 0 | 10 | 5.0 | 106 |
| Services booked at Patient Access Centre trough an office counter located in non-healthcare facilities | 3.1.1.96.5 | 1.5 | 0 | 10 | 3.6 | 233 |
| Services booked at Patient Access Centre trough an office counter located in local health facilities | 3.1.1.96.6 | 7.2 | 0 | 10 | 4.5 | 62 |
| Services booked at Patient Access Centre through web reservations | 3.1.1.96.7 | 5.2 | 0 | 10 | 5.0 | 96 |
| Credit/debit card at the Patient Access Centre office counter /other desks to pay services | 3.1.1.97.1 | 9.3 | 0 | 10 | 2.6 | 28 |
| Automatic cash machines at the facility to pay services | 3.1.1.97.2 | 3.7 | 0 | 10 | 4.8 | 129 |
| Shops to pay services outside the healthcare facility | 3.1.1.98.1 | 2.3 | 0 | 10 | 4.2 | 183 |
| Bank transfer or money order to pay services outside the healthcare facility | 3.1.1.98.2 | 6.3 | 0 | 10 | 4.8 | 77 |
| ATMs and/or other automatic cashiers to pay services outside the healthcare facility | 3.1.1.98.3 | 2.7 | 0 | 10 | 4.4 | 164 |
| Pharmacies to pay services outside the healthcare facility | 3.1.1.98.4 | 2.0 | 0 | 10 | 4.0 | 203 |
| Online payment to pay services outside the healthcare facility | 3.1.1.98.5 | 5.0 | 0 | 10 | 5.0 | 100 |
| One or more procedures to simplify the pre-operative clinical pathway | 3.1.2.99 | 9.7 | 0 | 10 | 1.8 | 18 |
| Reserved access to the Blood Tests and Samples Service for defined categories of users | 3.1.2.100 | 9.3 | 0 | 10 | 2.5 | 26 |
| Drugs delivered to continue the prescribed therapy for visited or discharged patients | 3.1.2.101.1 | 9.7 | 0 | 10 | 1.6 | 17 |
| Any documentations on drugs prescribed using a prescription pad charged to the NHS | 3.1.2.101.2 | 9.1 | 0 | 10 | 2.8 | 31 |
| Possibility to receive laboratory tests results at Home | 3.2.1.102.1 | 5.9 | 0 | 10 | 4.9 | 83 |
| Possibility to receive laboratory tests results Online | 3.2.1.102.2 | 6.2 | 0 | 10 | 4.9 | 79 |
| Possibility to receive the personal medical record at home | 3.2.1.103.1 | 9.1 | 0 | 10 | 2.9 | 32 |
| Possibility to consult the personal medical record online and to download it | 3.2.1.103.2 | 0.9 | 0 | 10 | 2.8 | 322 |
| Possibility to receive at home results of instrumental diagnostic tests | 3.2.1.104.1 | 5.6 | 0 | 10 | 5.0 | 88 |
| Online availability of results of instrumental diagnostic tests | 3.2.1.104.2 | 2.3 | 0 | 10 | 4.2 | 182 |
| Schedule diary to inform patients on their position in hospitalization waiting list of Orthopaedic Unit | 3.2.1.105 | 9.0 | 0 | 10 | 2.9 | 32 |
| Definition and communication of the maximum waiting time for the release of a copy of the medical record | 3.2.1.106 | 9.4 | 0 | 10 | 2.4 | 25 |
| Availability of digital images of ultrasound examinations | 3.2.1.107.1 | 7.5 | 0 | 10 | 4.3 | 57 |
| Availability of digital images of radiological exams | 3.2.1.107.2 | 10.0 | 0 | 10 | 0.7 | 7 |
| Copies of medical records available within 30 days of the request | 3.2.1.108 | 6.8 | 0 | 10 | 4.1 | 60 |
| Procedures/documentation on obligation for doctors to release the report of outpatient visits | 3.2.1.109 | 8.0 | 0 | 10 | 4.0 | 50 |
| Patient and public relation and engagement Office open >36 hours per week | 3.2.2.110.1 | 4.8 | 0 | 10 | 5.0 | 104 |
| Patient and public relation and engagement Office open 24 hours a day 1+ times a week | 3.2.2.110.2 | 5.8 | 0 | 10 | 5.0 | 85 |
| Patient and public relation and engagement Office open on Saturday | 3.2.2.110.3 | 2.8 | 0 | 10 | 4.5 | 160 |
| Patient and public relation and engagement Office open before 9AM 1+ times a week | 3.2.2.110.4 | 8.3 | 0 | 10 | 3.7 | 45 |
| Patient and public relation and engagement Office closed after 5PM 1+ times a week | 3.2.2.110.5 | 5.4 | 0 | 10 | 5.0 | 93 |
| Company/facility with a telephone number to provide information to the public | 3.2.2.111 | 7.3 | 0 | 10 | 2.8 | 39 |
| Brochures on diagnostic and care procedures for minors | 3.2.2.112 | 5.7 | 0 | 10 | 5.0 | 88 |
| 1+ information tools on doctor(s) in charge of the General Medicine Unit | 3.2.2.113.1 | 9.4 | 0 | 10 | 2.3 | 24 |
| 1+ information tools on the room of the doctor(s) receiving family at the General Medicine Unit | 3.2.2.113.2 | 8.9 | 0 | 10 | 3.2 | 36 |
| 1+ information tools on doctor(s) visiting hours at the General Medicine Unit | 3.2.2.113.3 | 9.5 | 0 | 10 | 2.2 | 23 |
| List of online services available at hospital website | 3.2.3.114.1 | 8.9 | 0 | 10 | 3.2 | 36 |
| List of online services offered by the Patient Access Centre at hospital website | 3.2.3.114.2 | 8.9 | 0 | 10 | 3.1 | 35 |
| List of e-participation tools available at hospital website | 3.2.3.114.3 | 4.4 | 0 | 10 | 5.0 | 112 |
| List of documents required to obtain medical records available at hospital website | 3.2.3.114.4 | 8.7 | 0 | 10 | 3.4 | 39 |
| Forms to request the medical record available at hospital website (download) | 3.2.3.114.5 | 7.3 | 0 | 10 | 4.4 | 61 |
| Telephone numbers and e-mail addresses available at hospital website | 3.2.3.114.6 | 9.3 | 0 | 10 | 2.5 | 27 |
| Waiting times for healthcare services available at hospital website | 3.2.3.114.7 | 7.7 | 0 | 10 | 4.2 | 54 |
| Presence at the hospital website of alternatives equivalent to audio/visual content | 3.2.3.115 | 2.6 | 0 | 10 | 4.4 | 171 |

**Table A1.4- Descriptive statistics - items AREA 4 (sd standard deviation; CV: coefficient of variation x 100)**

| **Description** | **Code** | **Mean** | **Min** | **Max** | **sd** | **CV** |
| --- | --- | --- | --- | --- | --- | --- |
| Presence of an operating procedure on informed consent | 4.1.1.116 | 9.3 | 0 | 10 | 2.5 | 27 |
| Presence of regular reports to monitor the application of the operating procedure on informed consent | 4.1.1.117 | 6.1 | 0 | 10 | 4.9 | 80 |
| Units with a defined welcome procedure | 4.1.1.118 | 6.2 | 0 | 10 | 4.6 | 75 |
| Units where each patient is assigned to 1+ healthcare professionals during care | 4.1.1.119 | 3.7 | 0 | 10 | 4.6 | 125 |
| Patient can have a free second opinion | 4.1.1.120 | 4.2 | 0 | 10 | 4.9 | 119 |
| Welcome service for citizens accessing the Emergency Room | 4.1.1.121 | 6.4 | 0 | 10 | 4.8 | 76 |
| Written protocol addressed to healthcare professionals to promote breastfeeding | 4.1.1.122 | 8.9 | 0 | 10 | 3.1 | 34 |
| 1+ education/information initiatives to promote breastfeeding among pregnant women | 4.1.1.123 | 9.7 | 0 | 10 | 1.7 | 18 |
| Organization of a Childbirth preparation course | 4.1.1.124 | 9.2 | 0 | 10 | 2.7 | 29 |
| Organization of a course on post-partum problems for new mothers/parents | 4.1.1.125 | 7.7 | 0 | 10 | 4.2 | 55 |
| 1+ initiatives of "health promotion" promoted with civic organizations | 4.1.1.126 | 6.7 | 0 | 10 | 4.1 | 61 |
| 1+ scientific/institutional/civic initiatives to encourage patients empowerment | 4.1.1.127 | 6.0 | 0 | 10 | 4.9 | 81 |
| 1+ initiatives to inform citizens on conservation/donation of hematopoietic stem cells from cord blood | 4.1.1.128 | 7.7 | 0 | 10 | 3.9 | 50 |
| 1+ initiative to inform on procedures to express willingness to donate organs | 4.1.1.129 | 7.5 | 0 | 10 | 3.8 | 51 |
| Activities/initiatives of narrative medicine | 4.1.1.130 | 2.6 | 0 | 10 | 3.8 | 151 |
| 1+ training on cultural diversity for health prof. in contact with foreigners last 36 mts | 4.1.2.131 | 6.0 | 0 | 10 | 4.9 | 82 |
| 1+ training for clinicians on clinical comm and/or helping relationship last 36 mts | 4.1.2.132.1 | 8.5 | 0 | 10 | 3.6 | 43 |
| 1+ training for nurses on clinical comm. and/or helping relationship last 36 mts | 4.1.2.132.2 | 8.5 | 0 | 10 | 3.6 | 43 |
| 1+ training course on end-of-life management for healthcare professionals | 4.1.2.133 | 7.5 | 0 | 10 | 4.4 | 59 |
| 1+ survey on organizational climate and well-being over the last 24 mts | 4.1.2.134.1 | 7.0 | 0 | 10 | 4.6 | 65 |
| 1+ improvement action on organizational climate and well-being after the survey | 4.1.2.134.2 | 5.3 | 0 | 10 | 5.0 | 95 |
| Service Charter available at the facility | 4.2.1.135.1 | 7.9 | 0 | 10 | 4.0 | 51 |
| Service Charter available at the website | 4.2.1.135.2 | 9.3 | 0 | 10 | 2.5 | 27 |
| Service Charter updated maximum 36 months ago | 4.2.1.136.1 | 8.8 | 0 | 10 | 3.3 | 38 |
| Service Charter including general information on services and procedures to access and use services | 4.2.1.136.2 | 9.4 | 0 | 10 | 2.4 | 26 |
| Service Charter includes section on commitments with related evaluation tools | 4.2.1.136.3 | 6.5 | 0 | 10 | 4.8 | 73 |
| Service Charter includes section on protection from inefficiency and limitations | 4.2.1.136.4 | 8.3 | 0 | 10 | 3.7 | 45 |
| Qualitative/quantitative analysis of complaints at Patient and public relation and engagement Office last 24 months | 4.2.1.137.1 | 9.3 | 0 | 10 | 2.6 | 28 |
| 1+ actions/measures following analysis of complaints | 4.2.1.137.2 | 8.4 | 0 | 10 | 3.7 | 44 |
| Survey on user satisfaction delivered during the last 24 months | 4.2.1.138.1 | 7.5 | 0 | 10 | 4.3 | 58 |
| Public disclosure of Results of survey on user satisfaction | 4.2.1.138.2 | 4.3 | 0 | 10 | 5.0 | 115 |
| 1+ improvement actions implemented following the survey on user satisfaction | 4.2.1.138.3 | 5.5 | 0 | 10 | 5.0 | 90 |
| Service charter of birth clinical pathway services drafted | 4.2.1.139 | 6.8 | 0 | 10 | 4.7 | 69 |
| Information point in the hall at the main entrance of the facility | 4.2.2.140 | 8.3 | 0 | 10 | 3.8 | 46 |
| Welcome service | 4.2.2.141 | 5.9 | 0 | 10 | 4.9 | 83 |
| 1+ training courses on relation/communication to users for front staff last 36 months | 4.2.3.142 | 6.5 | 0 | 10 | 4.8 | 74 |

**Table A2.1 – Factor loadings, item-total correlation, distribution of scores – AREA 1**

(N:responding hospitals;F1,F2,F3,F4: factor loadings; Std.r: item-total correlation) – Legend for F1-F4: Grey (Factor loading > 0.40, std.r >0.30): item attributed; White: item not attributed.

| **Summary Description** | **Code** | **N** | **F1** | **F2** | **F3** | **F4** | **std.r** | **Scores Distribution** | | | | | | | | | | | |
| --- | --- | --- | --- | --- | --- | --- | --- | --- | --- | --- | --- | --- | --- | --- | --- | --- | --- | --- | --- |
|  |  |  |  |  |  |  |  | **0** | **1** | **2** | **3** | **4** | **5** | **6** | **7** | **7.5** | **8** | **9** | **10** |
| Psychological support for cancer patients | 1.1.1.1 | 234 |  |  |  |  |  | 20 | 0 | 0 | 0 | 0 | 98 | 0 | 0 | 0 | 0 | 0 | 116 |
| Psychological support for victims of sexual violence | 1.1.1.2 | 283 | 0.34 | 0.23 | 0.17 | 0.17 | 0.48 | 26 | 0 | 0 | 0 | 0 | 65 | 0 | 0 | 0 | 0 | 0 | 192 |
| Psychological support for inpatients waiting/receiving organ transplant | 1.1.1.3 | 59 |  |  |  |  |  | 3 | 0 | 0 | 0 | 0 | 33 | 0 | 0 | 0 | 0 | 0 | 23 |
| Psychological support for children/adolescents in ordinary hospitalization | 1.1.1.4.1 | 206 |  |  |  |  |  | 31 | 0 | 0 | 0 | 0 | 26 | 0 | 0 | 104 | 0 | 0 | 45 |
| Psychological support for children/adolescents in Day hospital/Day Surgery | 1.1.1.4.2 | 190 |  |  |  |  |  | 31 | 0 | 0 | 0 | 0 | 27 | 0 | 0 | 95 | 0 | 0 | 37 |
| Psychological support for pregnancy-related problems | 1.1.1.5 | 226 |  |  |  |  |  | 20 | 0 | 0 | 0 | 0 | 95 | 0 | 0 | 0 | 0 | 0 | 111 |
| Projects to connect the hospital to the outside world | 1.1.2.6 | 387 | 0.37 | 0.18 | 0.13 | 0.19 | 0.48 | 124 | 0 | 0 | 0 | 0 | 80 | 0 | 0 | 22 | 0 | 0 | 161 |
| Projects to ensure educational needs/continuity at school | 1.1.2.7 | 125 |  |  |  |  |  | 42 | 0 | 0 | 0 | 0 | 0 | 0 | 0 | 0 | 0 | 0 | 83 |
| Projects to support playing and positive emotions in children | 1.1.2.8 | 210 |  |  |  |  |  | 16 | 0 | 0 | 0 | 0 | 26 | 0 | 0 | 7 | 0 | 0 | 161 |
| Visiting hours during weekdays | 1.1.2.9.1 | 387 | -0.14 | 0.1 | 0.17 | 0.51 | 0.32 | 155 | 0 | 0 | 0 | 0 | 136 | 0 | 0 | 0 | 0 | 0 | 96 |
| Visiting hours during weekends and holidays | 1.1.2.9.2 | 387 | -0.15 | 0.13 | 0.18 | 0.46 | 0.31 | 129 | 0 | 0 | 0 | 0 | 141 | 0 | 0 | 0 | 0 | 0 | 117 |
| Pregnant women can be supported by a trusted person in the labor room | 1.1.3.10.1 | 227 |  |  |  |  |  | 16 | 0 | 0 | 0 | 0 | 0 | 0 | 0 | 0 | 0 | 0 | 211 |
| Pregnant women can be supported by a trusted person in delivery room | 1.1.3.10.2 | 227 |  |  |  |  |  | 10 | 0 | 0 | 0 | 0 | 0 | 0 | 0 | 0 | 0 | 0 | 217 |
| Rooming-in available | 1.1.3.11 | 227 |  |  |  |  |  | 7 | 0 | 0 | 0 | 0 | 0 | 0 | 0 | 0 | 0 | 0 | 220 |
| Parent can assist own child in NICU without time limitation | 1.1.3.12 | 96 |  |  |  |  |  | 34 | 0 | 0 | 0 | 0 | 0 | 0 | 0 | 0 | 0 | 0 | 62 |
| Parent can assist own admitted child without time limitation | 1.1.3.13 | 210 |  |  |  |  |  | 7 | 0 | 0 | 0 | 0 | 4 | 0 | 0 | 44 | 0 | 0 | 155 |
| Parent can stay in pre-anaesthesia room/recovery room for invasive tests | 1.1.3.14.1 | 161 |  |  |  |  |  | 39 | 0 | 0 | 0 | 0 | 0 | 0 | 0 | 0 | 0 | 0 | 122 |
| Parent can stay in recovery room for invasive tests/surgery | 1.1.3.14.2 | 164 |  |  |  |  |  | 43 | 0 | 0 | 0 | 0 | 0 | 0 | 0 | 0 | 0 | 0 | 121 |
| Procedures on supplemental non-medical care | 1.1.3.15 | 387 | 0.17 | 0.08 | 0.00 | 0.30 | 0.34 | 85 | 0 | 0 | 0 | 0 | 0 | 0 | 0 | 0 | 0 | 0 | 302 |
| Open-Intensive-Care-Unit model is applied by extending visiting hours | 1.1.3.16 | 269 |  |  |  |  |  | 53 | 0 | 68 | 0 | 61 | 0 | 37 | 0 | 0 | 15 | 0 | 35 |
| Open-Intensive-Care-Unit model is applied by eliminating barrier devices | 1.1.3.17 | 269 |  |  |  |  |  | 130 | 0 | 0 | 0 | 0 | 0 | 0 | 0 | 0 | 0 | 0 | 139 |
| Agreements with accommodation facilities to offer reduced prices | 1.1.3.18 | 237 |  |  |  |  |  | 133 | 0 | 0 | 0 | 0 | 0 | 0 | 0 | 0 | 0 | 0 | 104 |
| Instruments for pain assessment available on medical record in General Medicine | 1.1.4.19.1 | 348 | 0.27 | 0.33 | 0.14 | -0.03 | 0.41 | 15 | 0 | 0 | 0 | 0 | 0 | 0 | 0 | 0 | 0 | 0 | 333 |
| Instruments for pain assessment available on medical record in General Surgery | 1.1.4.19.2 | 340 | 0.23 | 0.39 | 0.17 | -0.07 | 0.40 | 14 | 0 | 0 | 0 | 0 | 0 | 0 | 0 | 0 | 0 | 0 | 326 |
| Instruments for pain assessment available on medical record in Oncology | 1.1.4.19.3 | 235 |  |  |  |  |  | 7 | 0 | 0 | 0 | 0 | 0 | 0 | 0 | 0 | 0 | 0 | 228 |
| Guidelines/procedures for pain management adopted in General Medicine | 1.1.4.20.1 | 348 | 0.02 | 0.80 | 0.03 | 0.10 | 0.51 | 51 | 0 | 0 | 0 | 0 | 0 | 0 | 0 | 0 | 0 | 0 | 297 |
| Guidelines/procedures for pain management adopted in General Surgery | 1.1.4.20.2 | 340 | 0.02 | 0.76 | 0.10 | 0.10 | 0.51 | 39 | 0 | 0 | 0 | 0 | 0 | 0 | 0 | 0 | 0 | 0 | 301 |
| Guidelines/procedures for pain management adopted in Oncology | 1.1.4.20.3 | 235 |  |  |  |  |  | 23 | 0 | 0 | 0 | 0 | 0 | 0 | 0 | 0 | 0 | 0 | 212 |
| Guidelines/procedures for pain management adopted in Emergency Room | 1.1.4.20.4 | 298 | 0.18 | 0.68 | 0.08 | 0.14 | 0.58 | 50 | 0 | 0 | 0 | 0 | 0 | 0 | 0 | 0 | 0 | 0 | 248 |
| Labour analgesia guaranteed 24 hours a day with an anaesthesiologist | 1.1.4.21 | 227 |  |  |  |  |  | 73 | 0 | 0 | 0 | 0 | 29 | 0 | 0 | 0 | 0 | 0 | 125 |
| 1+ training courses on pain management for doctors last 36 months | 1.1.4.22.1 | 385 | 0.64 | 0.31 | -0.11 | -0.04 | 0.50 | 66 | 0 | 0 | 0 | 0 | 0 | 0 | 0 | 0 | 0 | 0 | 319 |
| 1+ training courses on pain management for nurses last 36 months | 1.1.4.22.2 | 385 | 0.64 | 0.32 | -0.10 | -0.02 | 0.50 | 65 | 0 | 0 | 0 | 0 | 0 | 0 | 0 | 0 | 0 | 0 | 320 |
| Information on post-surgery pain management | 1.1.4.23 | 340 | 0.14 | 0.43 | 0.16 | 0.09 | 0.43 | 60 | 0 | 0 | 0 | 0 | 0 | 0 | 0 | 0 | 0 | 0 | 280 |
| Activities to inform women about their right to give birth anonymously | 1.2.1.24 | 227 |  |  |  |  |  | 20 | 0 | 0 | 0 | 0 | 0 | 0 | 0 | 0 | 0 | 0 | 207 |
| Materials to communicate people accessing the ER they can nominate who can be informed | 1.2.1.25 | 298 | 0.20 | 0.32 | 0.07 | 0.14 | 0.41 | 62 | 0 | 0 | 0 | 0 | 0 | 0 | 0 | 0 | 0 | 0 | 236 |
| Materials to communicate people admitted to inpatient stay they can nominate who can be informed | 1.2.1.26 | 387 | 0.23 | 0.24 | 0.14 | 0.11 | 0.41 | 38 | 0 | 0 | 0 | 0 | 0 | 0 | 0 | 0 | 0 | 0 | 349 |
| Actions to prevent patients waiting at the blood tests and samples service from being called by name | 1.2.1.27 | 336 | 0.24 | 0.17 | 0.10 | 0.01 | 0.32 | 14 | 0 | 0 | 0 | 0 | 0 | 0 | 0 | 0 | 0 | 0 | 322 |
| Measures to limit the visibility of patients in ICU during visiting hours | 1.2.2.28 | 269 |  |  |  |  |  | 11 | 0 | 0 | 0 | 0 | 0 | 0 | 0 | 0 | 0 | 0 | 258 |
| Rooms reserved for terminal patients and family in General Medicine | 1.2.2.29 | 348 | 0.12 | 0.27 | 0.11 | 0.20 | 0.38 | 155 | 0 | 0 | 0 | 0 | 0 | 0 | 0 | 0 | 0 | 0 | 193 |
| Visual separation (partial or total) between beds in General Medicine | 1.2.2.30.1 | 348 | 0.14 | 0.11 | 0.79 | 0.10 | 0.43 | 63 | 22 | 24 | 30 | 15 | 6 | 8 | 4 | 0 | 5 | 8 | 163 |
| Visual separation (partial or total) between beds in General Surgery | 1.2.2.30.2 | 337 | 0.15 | 0.08 | 0.86 | 0.13 | 0.44 | 71 | 24 | 22 | 17 | 12 | 5 | 6 | 3 | 0 | 7 | 6 | 164 |
| Visual separation (partial or total) between beds in (specify) | 1.2.2.30.3 | 359 | 0.06 | 0.02 | 0.73 | 0.01 | 0.28 | 64 | 22 | 19 | 19 | 6 | 9 | 6 | 4 | 0 | 8 | 6 | 196 |
| Availability of an interpreter (onsite or on call) | 1.3.1.31 | 387 | 0.56 | 0.12 | 0.21 | 0.15 | 0.54 | 89 | 0 | 0 | 0 | 0 | 0 | 0 | 0 | 0 | 0 | 0 | 298 |
| 1+ forms of informed consent available in different languages | 1.3.1.32.1 | 387 | 0.34 | 0.16 | 0.07 | 0.44 | 0.53 | 222 | 0 | 0 | 0 | 0 | 0 | 0 | 0 | 0 | 0 | 0 | 165 |
| 1+ information sheets on available services (ED, obstetrics) | 1.3.1.32.2 | 387 | 0.44 | 0.13 | 0.12 | 0.40 | 0.57 | 171 | 0 | 0 | 0 | 0 | 0 | 0 | 0 | 0 | 0 | 0 | 216 |
| Service charter available in different languages | 1.3.1.32.3 | 387 | 0.31 | 0.21 | -0.03 | 0.33 | 0.46 | 230 | 0 | 0 | 0 | 0 | 0 | 0 | 0 | 0 | 0 | 0 | 157 |
| Discharge letter released in foreign language to requesting cross-border patients | 1.3.1.33 | 387 | 0.37 | 0.17 | 0.19 | 0.20 | 0.48 | 204 | 0 | 0 | 0 | 0 | 75 | 0 | 0 | 0 | 0 | 0 | 108 |
| Religious assistance for Catholics | 1.3.2.34 | 387 |  |  |  |  |  | 2 | 0 | 0 | 0 | 0 | 0 | 0 | 0 | 0 | 0 | 0 | 385 |
| Catholic Chapel available | 1.3.2.35.1 | 387 | 0.13 | 0.00 | -0.06 | 0.05 | 0.13 | 15 | 0 | 0 | 0 | 0 | 0 | 0 | 0 | 0 | 0 | 0 | 372 |
| Other areas dedicated to religious practice | 1.3.2.35.2 | 387 | 0.09 | 0.12 | 0.19 | 0.11 | 0.26 | 300 | 0 | 0 | 0 | 0 | 0 | 0 | 0 | 0 | 0 | 0 | 87 |
| Procedure for religious assistance for non-Catholics | 1.3.2.36 | 387 | 0.40 | 0.15 | 0.15 | 0.23 | 0.50 | 213 | 0 | 0 | 0 | 0 | 0 | 0 | 0 | 0 | 0 | 0 | 174 |
| Possibility to perform funerary functions according to ethical beliefs or faith (including body reconstruction by relatives). | 1.3.2.37 | 387 | 0.17 | 0.29 | 0.15 | 0.17 | 0.43 | 93 | 0 | 0 | 0 | 0 | 0 | 0 | 0 | 0 | 0 | 0 | 294 |
| Availability of services of cultural mediation (onsite or on call) | 1.3.3.38 | 387 | 0.56 | 0.01 | 0.14 | 0.17 | 0.47 | 118 | 0 | 0 | 0 | 0 | 0 | 0 | 0 | 0 | 0 | 0 | 269 |
| Projects to support use of services by users from other cultures | 1.3.3.39 | 387 | 0.50 | 0.03 | 0.16 | 0.15 | 0.45 | 209 | 0 | 0 | 0 | 0 | 35 | 0 | 0 | 17 | 0 | 0 | 126 |
| Possibility to choose or customise menu based on ethical preferences | 1.3.3.40.1 | 387 | 0.05 | 0.53 | -0.12 | 0.12 | 0.37 | 34 | 0 | 0 | 0 | 0 | 0 | 0 | 0 | 0 | 0 | 0 | 353 |
| Possibility to choose or customise menu based on religious preferences | 1.3.3.40.2 | 387 | 0.02 | 0.63 | -0.11 | 0.14 | 0.41 | 47 | 0 | 0 | 0 | 0 | 0 | 0 | 0 | 0 | 0 | 0 | 340 |
| Multilingual panel/poster/notice for STP code for foreign users (STP code) at Reception/information point/Patient and public relation and engagement Office | 1.3.3.41.1 | 387 | 0.16 | 0.20 | 0.00 | 0.66 | 0.53 | 230 | 0 | 0 | 0 | 0 | 0 | 0 | 0 | 0 | 0 | 0 | 157 |
| Multilingual panel/poster/notice for STP code for foreign users at Admission Office | 1.3.3.41.2 | 387 | 0.17 | 0.13 | 0.02 | 0.71 | 0.52 | 257 | 0 | 0 | 0 | 0 | 0 | 0 | 0 | 0 | 0 | 0 | 130 |
| Information leaflets on STP code for foreign users at 1+ Receptions | 1.3.3.41.3 | 387 | 0.24 | 0.08 | 0.00 | 0.65 | 0.51 | 243 | 0 | 0 | 0 | 0 | 0 | 0 | 0 | 0 | 0 | 0 | 144 |
| Website explaining how to obtain an STP code for foreign users | 1.3.3.41.4 | 387 | 0.25 | -0.04 | 0.02 | 0.36 | 0.35 | 177 | 0 | 0 | 0 | 0 | 0 | 0 | 0 | 0 | 0 | 0 | 210 |
| Multilingual printed notice at Point of Care indicating office and procedure for STP code | 1.3.3.41.5 | 387 | 0.26 | 0.10 | 0.04 | 0.63 | 0.54 | 254 | 0 | 0 | 0 | 0 | 0 | 0 | 0 | 0 | 0 | 0 | 133 |
| Designation of people supporting daily needs of patients using cross-border health care | 1.3.3.42 | 387 | 0.40 | 0.03 | 0.18 | 0.33 | 0.49 | 234 | 0 | 0 | 0 | 0 | 0 | 0 | 0 | 0 | 0 | 0 | 153 |
| Day Surgery discharge summary with details on who to refer to if needed | 1.4.1.43 | 375 | 0.22 | 0.15 | -0.04 | 0.02 | 0.24 | 14 | 0 | 0 | 0 | 0 | 0 | 0 | 0 | 0 | 0 | 0 | 361 |
| 1+ procedures to ensure continuity in the transition from another setting | 1.4.1.44 | 387 | 0.22 | 0.20 | 0.00 | 0.02 | 0.30 | 13 | 0 | 0 | 0 | 0 | 0 | 0 | 0 | 0 | 0 | 0 | 374 |
| Periodic audit reports on discharge with transition from another setting | 1.4.1.45 | 387 | 0.30 | 0.25 | -0.03 | 0.19 | 0.44 | 125 | 0 | 0 | 0 | 0 | 0 | 0 | 0 | 0 | 0 | 0 | 262 |
| Possibility to book post-discharge services through hospital doctor | 1.4.1.46 | 373 | 0.24 | 0.24 | 0.10 | 0.08 | 0.38 | 34 | 0 |  |  |  |  |  |  |  |  |  | 339 |
| Periodic reports on booking of post-hospitalization visits/exams | 1.4.1.47 | 370 | 0.19 | 0.29 | 0.05 | 0.13 | 0.39 | 204 | 0 |  |  |  |  |  |  |  |  |  | 166 |
| IT solution to share clinical data between facility and general practitioner | 1.4.1.48 | 387 | 0.27 | 0.01 | 0.09 | 0.09 | 0.29 | 293 | 0 | 0 |  |  |  |  |  |  |  |  | 94 |

**Table A2.2 – Factor loadings, item-total correlation, distribution of scores – AREA 2**

(N:responding hospitals;F1,F2,F3,F4: factor loadings; Std.r: item-total correlation) - Legend for F1-F4: Grey (Factor loading > 0.40, std.r >0.30): item attributed; White: item not attributed.

| **Description** | **Code** | **N** | **F1** | **F2** | **F3** | **F4** | **Std.r** | **Scores Distribution** | | | | | | | | | | | |
| --- | --- | --- | --- | --- | --- | --- | --- | --- | --- | --- | --- | --- | --- | --- | --- | --- | --- | --- | --- |
|  |  |  |  |  |  |  |  | **0** | **1** | **2** | **3** | **4** | **5** | **6** | **7** | **7.5** | **8** | **9** | **10** |
| Access for disabled to the Patient access centre through at least 1 pathway | 2.1.1.49.1 | 335 | 0.02 | 0.18 | -0.12 | 0.22 | 0.21 | 12 |  |  |  |  |  |  |  |  |  |  | 323 |
| Access for disabled to the Patient and public relation and engagement Office through at least 1 pathway | 2.1.1.49.2 | 308 | 0.05 | 0.25 | 0.00 | 0.20 | 0.33 | 13 |  |  |  |  |  |  |  |  |  |  | 295 |
| Access for disabled to the Imaging Service through at least 1pathway | 2.1.1.49.3 | 387 | 0.02 | 0.29 | -0.07 | 0.06 | 0.20 | 4 |  |  |  |  |  |  |  |  |  |  | 383 |
| Access for disabled to the Blood Tests and samples Service through at least 1pathways | 2.1.1.49.4 | 336 |  |  |  |  |  | 2 |  |  |  |  |  |  |  |  |  |  | 334 |
| Measures for access of blind/visually impaired to the Patient Access Centre | 2.1.1.50.1 | 335 | 0.86 | 0.09 | 0.14 | 0.08 | 0.46 | 277 |  |  |  |  |  |  |  |  |  |  | 58 |
| Measures for access of blind/visually impaired to the Patient and public relation and engagement Office | 2.1.1.50.2 | 308 | 0.90 | 0.10 | 0.05 | 0.12 | 0.45 | 255 |  |  |  |  |  |  |  |  |  |  | 53 |
| Measures for access of blind/visually impaired to the Imaging Service | 2.1.1.50.3 | 387 | 0.89 | 0.06 | 0.09 | 0.14 | 0.46 | 321 |  |  |  |  |  |  |  |  |  |  | 66 |
| Measures for access of blind/visually impaired to the Blood Tests and samples Service | 2.1.1.50.4 | 336 | 0.90 | 0.07 | 0.12 | 0.13 | 0.47 | 291 |  |  |  |  |  |  |  |  |  |  | 45 |
| Plan to remove architectural barriers | 2.1.1.51.1 | 251 |  |  |  |  |  | 124 |  |  |  |  |  |  |  |  |  |  | 127 |
| Plan to remove sensory barriers | 2.1.1.51.2 | 367 |  |  |  |  |  | 283 |  |  |  |  | 50 |  |  |  |  |  | 34 |
| Parking available for people accessing the facility | 2.1.2.52 | 385 | 0.00 | 0.04 | 0.07 | 0.20 | 0.22 | 20 |  |  |  |  | 170 |  |  |  |  |  | 195 |
| Area for cycles and/or motorcycles parking | 2.1.2.53 | 384 | 0.13 | 0.00 | 0.09 | 0.25 | 0.28 | 49 |  |  |  |  |  |  |  |  |  |  | 335 |
| Reserved parking slots for disabled people accessing the facility | 2.1.2.54 | 386 | -0.03 | -0.02 | -0.05 | 0.09 | 0.07 | 5 |  |  |  |  |  |  |  | 26 |  |  | 355 |
| Reserved parking slots for pregnant women ("pink parking") | 2.1.2.55 | 381 | 0.02 | 0.06 | -0.04 | 0.25 | 0.22 | 139 |  |  |  |  |  |  |  | 38 |  |  | 204 |
| 1 + public transport lines available to get to the hospital | 2.1.2.56 | 387 | -0.04 | -0.03 | 0.06 | 0.13 | 0.12 | 20 |  |  |  |  |  |  |  |  |  |  | 367 |
| Free bus shuttle service for users and visitors to move between pavilions of the facility | 2.1.2.57 | 61 |  |  |  |  |  | 39 |  |  |  |  |  |  |  |  |  |  | 22 |
| Taxi station close to the hospital, systems to call taxi, other tools | 2.1.2.58 | 387 | 0.07 | 0.19 | 0.05 | 0.45 | 0.44 | 115 |  |  |  |  |  |  |  |  |  |  | 272 |
| Possibility to stop vehicle in front of main entry for those with walk limitations | 2.1.2.59 | 386 |  |  |  |  |  | 4 |  |  |  |  |  |  |  |  |  |  | 382 |
| Internal pedestrian pathway or sidewalk | 2.1.2.60 | 325 |  |  |  |  |  | 20 |  |  |  |  |  |  |  |  |  |  | 305 |
| Main hall with 1+ updated signs indicating the location of the Patient Access Centre | 2.2.1.61.1 | 321 | -0.09 | 0.47 | 0.05 | 0.13 | 0.31 | 20 |  |  |  |  |  |  |  |  |  |  | 301 |
| Main hall with 1+ signs indicating the location of t the Patient and public relation and engagement Office | 2.2.1.61.2 | 302 | 0.01 | 0.48 | 0.13 | 0.19 | 0.43 | 34 |  |  |  |  |  |  |  |  |  |  | 268 |
| Main hall with 1+ signs indicating the location of the Imaging Service | 2.2.1.61.3 | 384 | 0.03 | 0.74 | 0.03 | 0.05 | 0.41 | 18 |  |  |  |  |  |  |  |  |  |  | 366 |
| Main hall with 1+ signs indicating the location of the General Medicine Unit | 2.2.1.61.4 | 348 | 0.04 | 0.91 | 0.04 | -0.05 | 0.41 | 22 |  |  |  |  |  |  |  |  |  |  | 326 |
| Main hall with 1+ signs indicating the location of the General Surgery Unit | 2.2.1.61.5 | 340 | 0.04 | 0.87 | 0.03 | -0.07 | 0.39 | 18 |  |  |  |  |  |  |  |  |  |  | 322 |
| Main hall with 1+ signs indicating the location of the General Direction | 2.2.1.61.6 | 365 | 0.03 | 0.68 | 0.11 | 0.04 | 0.41 | 31 |  |  |  |  |  |  |  |  |  |  | 334 |
| Internal signs showing pathways for different destination areas | 2.2.1.62 | 387 | 0.18 | 0.25 | 0.11 | 0.24 | 0.41 | 98 |  |  |  |  |  |  |  |  |  |  | 289 |
| Internal pathways allowing inpatients of General Surgery Unit to reach operating room | 2.2.2.63.1 | 340 | 0.06 | 0.17 | 0.00 | 0.22 | 0.27 | 20 |  |  |  |  |  |  |  |  |  |  | 320 |
| Internal pathways allowing pregnant women to reach the delivery/operating room | 2.2.2.63.2 | 227 |  |  |  |  |  | 7 |  |  |  |  |  |  |  |  |  |  | 220 |
| Internal pathways allowing inpatients to reach the Imaging Service (without passing through the waiting room) | 2.2.2.64 | 387 | 0.17 | 0.23 | 0.08 | 0.24 | 0.40 | 116 |  |  |  |  |  |  |  |  |  |  | 271 |
| Personal use of television in rooms of General Medicine | 2.3.1.65.1 | 341 | 0.07 | 0.08 | 0.44 | 0.24 | 0.42 | 207 | 12 | 8 | 10 | 2 | 5 | 4 | 1 |  | 2 | 4 | 86 |
| Personal use of television in rooms of General Surgery | 2.3.1.65.2 | 333 | 0.06 | 0.05 | 0.47 | 0.18 | 0.39 | 189 | 15 | 8 | 7 | 2 | 3 | 1 |  |  | 2 | 4 | 102 |
| Personal use of television in rooms of the Unit (specify) ………… | 2.3.1.65.3 | 356 | 0.01 | 0.06 | 0.43 | 0.20 | 0.35 | 172 | 14 | 3 | 7 | 3 | 6 | 8 | 1 |  | 3 | 2 | 137 |
| Rooms with air conditioning system in General Medicine | 2.3.1.66.1 | 344 | -0.02 | 0.01 | 0.69 | -0.08 | 0.29 | 42 | 8 | 1 | 3 | 1 | 4 | 2 |  |  |  | 3 | 280 |
| Rooms with air conditioning in General Surgery | 2.3.1.66.2 | 336 | 0.01 | 0.08 | 0.77 | -0.10 | 0.36 | 38 | 3 | 1 | 1 | 4 | 5 | 2 |  |  | 1 | 0 | 281 |
| Rooms with air conditioning in the Unit (specify)………… | 2.3.1.66.3 | 357 | -0.01 | -0.10 | 0.59 | -0.12 | 0.18 | 26 | 2 | 1 |  | 2 | 3 | 1 | 2 |  | 1 | 1 | 318 |
| Rooms with no more than 2 beds in the General Medicine Unit | 2.3.1.67.1 | 342 | 0.16 | 0.01 | 0.24 | 0.16 | 0.31 | 23 | 13 | 20 | 34 | 35 | 25 | 21 | 16 |  | 19 | 18 | 118 |
| Rooms with no more than 2 beds in the General Surgery Unit | 2.3.1.67.2 | 334 | 0.13 | -0.03 | 0.22 | 0.16 | 0.27 | 23 | 13 | 17 | 32 | 25 | 19 | 24 | 21 |  | 13 | 21 | 126 |
| Rooms with no more than 2 beds in the Unit (specify)………… | 2.3.1.67.3 | 357 | 0.16 | 0.05 | 0.19 | 0.14 | 0.30 | 30 | 12 | 11 | 26 | 20 | 16 | 16 | 14 |  | 20 | 17 | 175 |
| Free internet access via wireless network | 2.3.1.68 | 387 | 0.17 | 0.08 | 0.10 | 0.41 | 0.41 | 218 |  |  |  |  |  |  |  |  |  |  | 169 |
| Rooms with internal bathrooms in the General Medicine Unit | 2.3.1.69.1 | 344 | 0.02 | -0.05 | 0.44 | -0.05 | 0.19 | 21 | 14 | 6 | 8 | 6 | 7 | 3 | 6 |  | 3 | 11 | 259 |
| Rooms with internal bathrooms in the General Surgery Unit | 2.3.1.69.2 | 335 | 0.06 | -0.04 | 0.45 | -0.10 | 0.19 | 20 | 11 | 5 | 8 | 3 | 5 | 3 | 5 |  | 2 | 8 | 265 |
| Rooms with internal bathrooms in the Unit (specify)………… | 2.3.1.69.3 | 357 | 0.07 | 0.02 | 0.43 | 0.04 | 0.30 | 20 | 4 | 7 | 9 | 3 | 8 | 5 | 4 |  | 5 | 7 | 285 |
| Dining room reserved for patients and their families in the Psychiatric Unit | 2.3.1.70 | 172 |  |  |  |  |  | 23 |  |  |  |  |  |  |  |  |  |  | 149 |
| Sitting room or any other area for social activities in the Psychiatric Unit | 2.3.1.71 | 172 |  |  |  |  |  | 24 |  |  |  |  |  |  |  |  |  |  | 148 |
| Paediatric Units equipped with play area | 2.3.2.72.1 | 210 |  |  |  |  |  | 13 | 1 | 1 | 2 |  | 1 |  |  |  | 2 | 3 | 187 |
| Paediatric Units equipped with reading corners/ library | 2.3.2.72.2 | 209 |  |  |  |  |  | 26 | 3 | 2 | 1 |  | 3 |  |  |  |  | 2 | 172 |
| Paediatric Units equipped with furniture suitable for children | 2.3.2.73.1 | 210 |  |  |  |  |  | 13 |  |  | 1 |  | 1 |  |  |  | 1 |  | 194 |
| Paediatric Units equipped with coloured walls and corridors | 2.3.2.73.2 | 210 |  |  |  |  |  | 8 |  |  | 1 |  | 1 |  |  |  |  |  | 200 |
| Paediatric Units equipped with specific decorations on the walls | 2.3.2.73.3 | 210 |  |  |  |  |  | 9 |  |  | 1 |  | 1 |  |  |  |  | 1 | 198 |
| Waiting room dedicated for children in the Emergency room | 2.3.2.74 | 143 |  |  |  |  |  | 51 |  |  |  |  |  |  |  |  |  |  | 92 |
| Child-friendly operating room | 2.3.2.75 | 67 |  |  |  |  |  | 37 |  |  |  |  |  |  |  |  |  |  | 30 |
| Possibility for patients on a free diet to choose 2+ menu options | 2.3.3.76 | 387 | 0.01 | -0.01 | -0.03 | 0.21 | 0.15 | 11 |  |  |  |  |  |  |  |  |  |  | 376 |
| Breakfast time | 2.3.3.77 | 387 |  |  |  |  |  | 6 |  |  |  |  |  |  |  |  |  |  | 381 |
| Lunch time | 2.3.3.78 | 387 | 0.11 | -0.03 | 0.02 | 0.08 | 0.14 | 41 |  |  |  |  |  |  |  |  |  |  | 346 |
| Dinner time | 2.3.3.79 | 387 | 0.05 | 0.01 | -0.03 | 0.14 | 0.15 | 281 |  |  |  |  |  |  |  |  |  |  | 106 |
| Obtaining extra cleaning services on demand by patient and/or health professionals | 2.3.3.80 | 387 | 0.02 | 0.26 | 0.08 | 0.17 | 0.30 | 10 |  |  |  |  |  |  |  |  |  |  | 377 |
| Staff name/code and timing of daily bathroom cleaning shown in Emergency Room | 2.3.3.81.1 | 298 | 0.18 | 0.21 | -0.01 | 0.37 | 0.42 | 160 |  |  |  |  |  |  |  |  |  |  | 138 |
| Staff name/code and timing of daily bathroom cleaning shown in Patient Access Centre | 2.3.3.81.2 | 335 | 0.14 | 0.20 | 0.00 | 0.46 | 0.45 | 183 |  |  |  |  |  |  |  |  |  |  | 152 |
| Patients can receive meal and/or food during visit in Emergency Room | 2.3.3.82.1 | 298 | 0.11 | 0.07 | 0.00 | 0.23 | 0.26 | 121 |  |  |  |  |  |  |  |  |  |  | 177 |
| Patients can receive meal and/or food during visit in Outpatient Clinic | 2.3.3.82.2 | 356 | 0.08 | 0.18 | -0.02 | 0.21 | 0.26 | 45 |  |  |  |  |  |  |  |  |  |  | 311 |
| Inpatients and their family can buy newspapers and magazines | 2.4.1.83 | 387 | -0.03 | 0.07 | 0.07 | 0.57 | 0.42 | 134 |  |  |  |  |  |  |  |  |  |  | 253 |
| Inpatients and their family can buy essential accessories for personal care | 2.4.1.84 | 387 | 0.04 | 0.12 | 0.12 | 0.53 | 0.47 | 145 |  |  |  |  |  |  |  |  |  |  | 242 |
| ATM inside the facility | 2.4.1.85 | 387 | 0.06 | -0.05 | 0.03 | 0.35 | 0.28 | 185 |  |  |  |  |  |  |  |  |  |  | 202 |
| Safety deposit service for inpatients | 2.4.1.86 | 387 | 0.13 | 0.14 | -0.05 | 0.39 | 0.36 | 178 |  |  |  |  | 93 |  |  |  |  |  | 116 |
| Barber/hairdresser available inside facility or on call | 2.4.1.87 | 387 | 0.03 | 0.13 | 0.03 | 0.35 | 0.32 | 188 |  |  |  |  | 60 |  |  |  |  |  | 139 |
| Coffee bar | 2.4.1.88 | 387 | -0.06 | 0.11 | 0.13 | 0.52 | 0.41 | 112 |  |  |  |  |  |  |  |  |  |  | 275 |
| Reading corner with books/magazines freely available to inpatients/family | 2.4.1.89 | 387 | 0.16 | 0.07 | 0.05 | 0.49 | 0.44 | 180 |  |  |  |  | 91 |  |  |  |  |  | 116 |
| Green area / garden with benches accessible to inpatients and family | 2.4.1.90 | 387 | -0.04 | 0.07 | 0.22 | 0.29 | 0.35 | 110 |  |  |  |  | 21 |  |  |  |  |  | 256 |
| Adequate dedicated space in the Waiting room of the Emergency Department | 2.4.2.91.1 | 298 |  |  |  |  |  | 11 |  |  |  |  |  |  |  |  |  |  | 287 |
| Seats available in the waiting room of the Emergency Department | 2.4.2.91.2 | 298 |  |  |  |  |  | 0 |  |  |  |  |  |  |  |  |  |  | 298 |
| Food vending machines available in the Waiting room of Emergency Department | 2.4.2.91.3 | 298 |  |  |  |  |  | 26 |  |  |  |  |  |  |  |  |  |  | 272 |
| Air conditioning available in the waiting room of Emergency Department | 2.4.2.91.4 | 298 | 0.04 | 0.15 | 0.43 | 0.08 | 0.37 | 22 |  |  |  |  |  |  |  |  |  |  | 276 |
| Display counter available in the waiting room of Emergency Department with real time number of patients waiting | 2.4.2.91.5 | 263 |  |  |  |  |  | 80 |  |  |  |  |  |  |  |  |  |  | 183 |
| Television available in the waiting room of Emergency Department | 2.4.2.91.6 | 298 | 0.05 | 0.08 | 0.14 | 0.32 | 0.36 | 126 |  |  |  |  |  |  |  |  |  |  | 172 |
| Adequate dedicated space in the Waiting room of Blood Tests and samples Service | 2.4.2.92.1 | 336 | 0.04 | 0.03 | 0.12 | 0.21 | 0.26 | 39 |  |  |  |  |  |  |  |  |  |  | 297 |
| Seats available in the waiting room of Blood Tests and samples Service | 2.4.2.92.2 | 336 | 0.02 | -0.01 | 0.04 | 0.20 | 0.20 | 7 |  |  |  |  |  |  |  |  |  |  | 329 |
| Food vending machines available in the Waiting room of Blood Tests and samples Service | 2.4.2.92.3 | 336 | 0.08 | 0.13 | 0.04 | 0.22 | 0.28 | 61 |  |  |  |  |  |  |  |  |  |  | 275 |
| Air conditioning available in the waiting room of Blood Test and samples Service | 2.4.2.92.4 | 336 | 0.01 | 0.12 | 0.46 | 0.18 | 0.43 | 42 |  |  |  |  |  |  |  |  |  |  | 294 |
| Adequate dedicated space in the Waiting room of Intensive Care | 2.4.2.93.1 | 269 |  |  |  |  |  | 63 |  |  |  |  |  |  |  |  |  |  | 206 |
| Seats available in the waiting room of Intensive Care | 2.4.2.93.2 | 269 |  |  |  |  |  | 19 |  |  |  |  |  |  |  |  |  |  | 250 |
| Food vending machines available in the Waiting room of Intensive Care | 2.4.2.93.3 | 269 |  |  |  |  |  | 81 |  |  |  |  |  |  |  |  |  |  | 188 |
| Air conditioning available in the waiting room of Intensive Care | 2.4.2.93.4 | 269 |  |  |  |  |  | 44 |  |  |  |  |  |  |  |  |  |  | 225 |
| Adequate dedicated space in the Waiting room of Delivery Room | 2.4.2.94.1 | 227 |  |  |  |  |  | 70 |  |  |  |  |  |  |  |  |  |  | 157 |
| Seats available in the waiting room of Delivery Room | 2.4.2.94.2 | 227 |  |  |  |  |  | 23 |  |  |  |  |  |  |  |  |  |  | 204 |
| Food vending machines available in the Waiting room of Delivery Room | 2.4.2.94.3 | 227 |  |  |  |  |  | 75 |  |  |  |  |  |  |  |  |  |  | 152 |
| Air conditioning available in the waiting room of Delivery Room | 2.4.2.94.4 | 227 |  |  |  |  |  | 46 |  |  |  |  |  |  |  |  |  |  | 181 |

**Table A2.3 – Factor loadings, item-total correlation, distribution of scores – AREA 3**

(N:responding hospitals;F1,F2,F3,F4: factor loadings; Std.r: item-total correlation) - Legend for F1-F4: Grey (Factor loading > 0.40, std.r >0.30): item attributed; White: item not attributed.

| **Description** | **Code** | **N** | **F1** | **F2** | **F3** | **F4** | **Std.r** | **Scores Distribution** | | | | | | | | | | | |
| --- | --- | --- | --- | --- | --- | --- | --- | --- | --- | --- | --- | --- | --- | --- | --- | --- | --- | --- | --- |
|  |  |  |  |  |  |  |  | **0** | **1** | **2** | **3** | **4** | **5** | **6** | **7** | **7.5** | **8** | **9** | **10** |
| Health services booked at 1+ office counter of Patient Access Centre open >36hrs per week | 3.1.1.95.1 | 387 | 0.03 | 0.66 | 0.10 | 0.29 | 0.38 | 98 |  |  |  |  |  |  |  |  |  |  | 289 |
| Health services booked at 1+ office counter of Patient Access Centre open all day 1+ days per week | 3.1.1.95.2 | 387 | 0.09 | 0.64 | 0.04 | 0.21 | 0.41 | 118 |  |  |  |  |  |  |  |  |  |  | 269 |
| Health services booked at 1+ desks of Patient Access Centre open on Saturdays | 3.1.1.95.3 | 387 | -0.17 | 0.61 | 0.09 | 0.30 | 0.26 | 143 |  |  |  |  |  |  |  |  |  |  | 244 |
| Health services booked at 1+ office counter of Patient Access Centre before 9AM 1+ days per week | 3.1.1.95.4 | 387 | -0.07 | 0.44 | 0.32 | -0.03 | 0.31 | 25 |  |  |  |  |  |  |  |  |  |  | 362 |
| Health services booked at 1+ office counter of Patient Access Centre closing 5PM 1+ days per week | 3.1.1.95.5 | 387 | -0.17 | 0.51 | 0.10 | 0.31 | 0.19 | 133 |  |  |  |  |  |  |  |  |  |  | 254 |
| Services booked at Patient Access Centre at a office counter with dedicated operator | 3.1.1.96.1 | 387 | 0.24 | 0.10 | 0.26 | 0.08 | 0.35 | 42 |  |  |  |  |  |  |  |  |  |  | 345 |
| Services booked at the Patient Access Centre by phone | 3.1.1.96.2 | 387 | 0.12 | 0.23 | 0.08 | -0.02 | 0.30 | 19 |  |  |  |  |  |  |  |  |  |  | 368 |
| Services booked at Patient Access Centre through the General Practitioner | 3.1.1.96.3 | 387 | 0.09 | 0.15 | 0.34 | 0.07 | 0.33 | 262 |  |  |  |  |  |  |  |  |  |  | 125 |
| Services booked at Patient Access Centre through a Pharmacy | 3.1.1.96.4 | 387 | 0.04 | 0.04 | 0.55 | -0.01 | 0.32 | 205 |  |  |  |  |  |  |  |  |  |  | 182 |
| Services booked at Patient Access Centre trough an office counter located in non-healthcare facilities | 3.1.1.96.5 | 387 | 0.12 | 0.00 | 0.28 | 0.03 | 0.24 | 327 |  |  |  |  |  |  |  |  |  |  | 60 |
| Services booked at Patient Access Centre trough an office counter located in local health facilities | 3.1.1.96.6 | 387 | 0.19 | 0.03 | 0.44 | -0.07 | 0.34 | 108 |  |  |  |  |  |  |  |  |  |  | 279 |
| Services booked at Patient Access Centre through web reservations | 3.1.1.96.7 | 387 | 0.23 | 0.09 | 0.17 | -0.03 | 0.34 | 186 |  |  |  |  |  |  |  |  |  |  | 201 |
| Credit/debit card at the Patient Access Centre office counter /other desks to pay services | 3.1.1.97.1 | 387 | 0.18 | 0.23 | 0.01 | -0.11 | 0.30 | 29 |  |  |  |  |  |  |  |  |  |  | 358 |
| Automatic cash machines at the facility to pay services | 3.1.1.97.2 | 387 | 0.47 | 0.02 | 0.23 | -0.24 | 0.46 | 242 |  |  |  |  |  |  |  |  |  |  | 145 |
| Shops to pay services outside the healthcare facility | 3.1.1.98.1 | 387 | -0.37 | -0.06 | 0.15 | -0.15 | 0.33 | 298 |  |  |  |  |  |  |  |  |  |  | 89 |
| Bank transfer or money order to pay services outside the healthcare facility | 3.1.1.98.2 | 387 | 0.07 | -0.02 | 0.15 | -0.15 | 0.14 | 143 |  |  |  |  |  |  |  |  |  |  | 244 |
| ATMs and/or other automatic cashiers to pay services outside the healthcare facility | 3.1.1.98.3 | 387 | -0.09 | -0.05 | 0.28 | -0.13 | 0.06 | 282 |  |  |  |  |  |  |  |  |  |  | 105 |
| Pharmacies to pay services outside the healthcare facility | 3.1.1.98.4 | 387 | -0.13 | 0.14 | 0.43 | -0.18 | 0.16 | 311 |  |  |  |  |  |  |  |  |  |  | 76 |
| Online payment to pay services outside the healthcare facility | 3.1.1.98.5 | 387 | 0.35 | 0.02 | 0.29 | -0.21 | 0.42 | 193 |  |  |  |  |  |  |  |  |  |  | 194 |
| One or more procedures to simplify the pre-operative clinical pathway | 3.1.2.99 | 363 | 0.16 | 0.09 | 0.00 | 0.11 | 0.24 | 12 |  |  |  |  |  |  |  |  |  |  | 351 |
| Reserved access to the Blood Tests and Samples Service for defined categories of users | 3.1.2.100 | 336 | 0.14 | 0.18 | 0.07 | 0.09 | 0.28 | 22 |  |  |  |  |  |  |  |  |  |  | 314 |
| Drugs delivered to continue the prescribed therapy for visited or discharged patients | 3.1.2.101.1 | 357 | 0.12 | 0.06 | 0.01 | -0.17 | 0.17 | 10 |  |  |  |  |  |  |  |  |  |  | 347 |
| Any documentations on drugs prescribed using a prescription pad charged to the NHS | 3.1.2.101.2 | 338 | 0.01 | 0.33 | -0.06 | -0.06 | 0.19 | 29 |  |  |  |  |  |  |  |  |  |  | 309 |
| Possibility to receive laboratory tests results at Home | 3.2.1.102.1 | 336 | 0.62 | 0.27 | -0.16 | 0.13 | 0.51 | 136 |  |  |  |  |  |  |  |  |  |  | 200 |
| Possibility to receive laboratory tests results Online | 3.2.1.102.2 | 336 | 0.40 | 0.31 | 0.04 | -0.20 | 0.43 | 128 |  |  |  |  |  |  |  |  |  |  | 208 |
| Possibility to receive the personal medical record at home | 3.2.1.103.1 | 387 | 0.42 | 0.05 | -0.04 | -0.08 | 0.34 | 35 |  |  |  |  |  |  |  |  |  |  | 352 |
| Possibility to consult the personal medical record online and to download it | 3.2.1.103.2 | 387 | 0.16 | -0.09 | 0.02 | 0.01 | 0.12 | 353 |  |  |  |  |  |  |  |  |  |  | 34 |
| Possibility to receive at home results of instrumental diagnostic tests | 3.2.1.104.1 | 383 | 0.55 | 0.22 | -0.13 | 0.13 | 0.46 | 167 |  |  |  |  |  |  |  |  |  |  | 216 |
| Online availability of results of instrumental diagnostic tests | 3.2.1.104.2 | 384 | 0.38 | 0.16 | 0.00 | -0.08 | 0.36 | 295 |  |  |  |  |  |  |  |  |  |  | 89 |
| Schedule diary to inform patients on their position in hospitalization waiting list of Orthopaedic Unit | 3.2.1.105 | 326 | 0.08 | 0.39 | 0.04 | 0.05 | 0.29 | 31 |  |  |  |  |  |  |  |  |  |  | 295 |
| Definition and communication of the maximum waiting time for the release of a copy of the medical record | 3.2.1.106 | 387 | -0.07 | 0.07 | -0.01 | 0.15 | 0.06 | 23 |  |  |  |  |  |  |  |  |  |  | 364 |
| Availability of digital images of ultrasound examinations | 3.2.1.107.1 | 382 | 0.00 | 0.00 | -0.08 | 0.10 | 0.11 | 94 |  |  |  |  |  |  |  |  |  |  | 288 |
| Availability of digital images of radiological exams | 3.2.1.107.2 | 384 |  |  |  |  |  | 2 |  |  |  |  |  |  |  |  |  |  | 382 |
| Copies of medical records available within 30 days of the request | 3.2.1.108 | 387 | 0.23 | 0.11 | 0.02 | -0.04 | 0.27 | 87 | 3 | 2 | 8 | 5 | 10 | 12 | 18 |  | 23 | 53 | 166 |
| Procedures/documentation on obligation for doctors to release the report of outpatient visits | 3.2.1.109 | 380 | 0.17 | -0.06 | 0.01 | 0.14 | 0.20 | 75 |  |  |  |  |  |  |  |  |  |  | 305 |
| Patient and public relation and engagement Office open >36 hours per week | 3.2.2.110.1 | 280 | 0.22 | 0.12 | -0.02 | 0.66 | 0.27 | 145 |  |  |  |  |  |  |  |  |  |  | 135 |
| Patient and public relation and engagement Office open 24 hours a day 1+ times a week | 3.2.2.110.2 | 281 | 0.24 | 0.10 | 0.01 | 0.50 | 0.32 | 117 |  |  |  |  |  |  |  |  |  |  | 164 |
| Patient and public relation and engagement Office open on Saturday | 3.2.2.110.3 | 283 | 0.02 | 0.16 | 0.14 | 0.60 | 0.25 | 203 |  |  |  |  |  |  |  |  |  |  | 80 |
| Patient and public relation and engagement Office open before 9AM 1+ times a week | 3.2.2.110.4 | 284 | 0.07 | 0.07 | -0.04 | 0.33 | 0.17 | 47 |  |  |  |  |  |  |  |  |  |  | 237 |
| Patient and public relation and engagement Office closed after 5PM 1+ times a week | 3.2.2.110.5 | 284 | -0.11 | 0.00 | 0.00 | 0.54 | 0.05 | 131 |  |  |  |  |  |  |  |  |  |  | 153 |
| Company/facility with a telephone number to provide information to the public | 3.2.2.111 | 387 | -0.06 | -0.12 | 0.17 | 0.14 | 0.08 | 13 |  |  |  |  | 185 |  |  |  |  |  | 189 |
| Brochures on diagnostic and care procedures for minors | 3.2.2.112 | 210 |  |  |  |  |  | 91 |  |  |  |  |  |  |  |  |  |  | 119 |
| 1+ information tools on doctor(s) in charge of the General Medicine Unit | 3.2.2.113.1 | 348 | 0.12 | 0.03 | 0.18 | 0.04 | 0.25 | 19 |  |  |  |  |  |  |  |  |  |  | 329 |
| 1+ information tools on the room of the doctor(s) receiving family at the General Medicine Unit | 3.2.2.113.2 | 348 | -0.04 | 0.05 | 0.24 | 0.06 | 0.15 | 39 |  |  |  |  |  |  |  |  |  |  | 309 |
| 1+ information tools on doctor(s) visiting hours at the General Medicine Unit | 3.2.2.113.3 | 348 | 0.09 | 0.04 | 0.25 | -0.11 | 0.24 | 17 |  |  |  |  |  |  |  |  |  |  | 331 |
| List of online services available at hospital website | 3.2.3.114.1 | 387 | 0.49 | -0.20 | 0.15 | 0.03 | 0.36 | 44 |  |  |  |  |  |  |  |  |  |  | 343 |
| List of online services offered by the Patient Access Centre at hospital website | 3.2.3.114.2 | 387 | 0.38 | -0.14 | 0.31 | -0.08 | 0.36 | 42 |  |  |  |  |  |  |  |  |  |  | 345 |
| List of e-participation tools available at hospital website | 3.2.3.114.3 | 387 | 0.30 | -0.01 | 0.31 | 0.18 | 0.37 | 215 |  |  |  |  |  |  |  |  |  |  | 172 |
| List of documents required to obtain medical records available at hospital website | 3.2.3.114.4 | 387 | 0.54 | -0.12 | 0.17 | 0.01 | 0.40 | 52 |  |  |  |  |  |  |  |  |  |  | 335 |
| Forms to request the medical record available at hospital website (download) | 3.2.3.114.5 | 387 | 0.58 | -0.06 | 0.18 | 0.01 | 0.47 | 104 |  |  |  |  |  |  |  |  |  |  | 283 |
| Telephone numbers and e-mail addresses available at hospital website | 3.2.3.114.6 | 387 | 0.27 | 0.11 | 0.12 | -0.02 | 0.37 | 26 |  |  |  |  |  |  |  |  |  |  | 361 |
| Waiting times for healthcare services available at hospital website | 3.2.3.114.7 | 387 | 0.24 | -0.04 | 0.10 | -0.26 | 0.21 | 87 |  |  |  |  |  |  |  |  |  |  | 300 |
| Presence at the hospital website of alternatives equivalent to audio/visual content | 3.2.3.115 | 387 | 0.42 | -0.03 | 0.17 | -0.05 | 0.39 | 288 |  |  |  |  |  |  |  |  |  |  | 99 |

**Table A2.4 – Factor loadings, item-total correlation, distribution of scores – AREA 4**

(N:responding hospitals;F1,F2,F3,F4: factor loadings; Std.r: item-total correlation) - Legend for F1-F4: Grey (Factor loading > 0.40, std.r >0.30): item attributed; White: item not attributed.

| **Description** | **Code** | **N** | **F1** | **F2** | **F3** | **F4** | **Std.r** | **Scores Distribution** | | | | | | | | | | | |
| --- | --- | --- | --- | --- | --- | --- | --- | --- | --- | --- | --- | --- | --- | --- | --- | --- | --- | --- | --- |
|  |  |  |  |  |  |  |  | **0** | **1** | **2** | **3** | **4** | **5** | **6** | **7** | **7.5** | **8** | **9** | **10** |
| Presence of an operating procedure on informed consent | 4.1.1.116 | 387 | 0.16 | 0.04 | 0.18 | 0.31 | 0.38 | 26 |  |  |  |  |  |  |  |  |  |  | 361 |
| Presence of regular reports to monitor the application of the operating procedure on informed consent | 4.1.1.117 | 387 | 0.21 | 0.14 | 0.14 | 0.49 | 0.51 | 151 |  |  |  |  |  |  |  |  |  |  | 236 |
| Units with a defined welcome procedure | 4.1.1.118 | 387 | 0.17 | 0.19 | 0.19 | 0.49 | 0.54 | 125 | 6 | 4 | 5 | 4 | 6 | 4 | 5 |  | 8 | 8 | 212 |
| Units where each patient is assigned to 1+ healthcare professionals during care | 4.1.1.119 | 387 | 0.08 | 0.06 | 0.13 | 0.45 | 0.39 | 205 | 16 | 15 | 10 | 3 | 8 | 2 | 1 |  |  |  | 127 |
| Patient can have a free second opinion | 4.1.1.120 | 387 | 0.19 | 0.20 | 0.00 | 0.26 | 0.37 | 226 |  |  |  |  |  |  |  |  |  |  | 161 |
| Welcome service for citizens accessing the Emergency Room | 4.1.1.121 | 171 |  |  |  |  |  | 62 |  |  |  |  |  |  |  |  |  |  | 109 |
| Written protocol addressed to healthcare professionals to promote breastfeeding | 4.1.1.122 | 227 |  |  |  |  |  | 24 |  |  |  |  |  |  |  |  |  |  | 203 |
| 1+ education/information initiatives to promote breastfeeding among pregnant women | 4.1.1.123 | 227 |  |  |  |  |  | 7 |  |  |  |  |  |  |  |  |  |  | 220 |
| Organization of a Childbirth preparation course | 4.1.1.124 | 227 |  |  |  |  |  | 18 |  |  |  |  |  |  |  |  |  |  | 209 |
| Organization of a course on post-partum problems for new mothers/parents | 4.1.1.125 | 227 |  |  |  |  |  | 53 |  |  |  |  |  |  |  |  |  |  | 174 |
| 1+ initiatives of "health promotion" promoted with civic organizations | 4.1.1.126 | 387 | 0.21 | 0.54 | 0.10 | 0.22 | 0.57 | 90 |  |  |  |  | 64 |  |  | 16 |  |  | 217 |
| 1+ scientific/institutional/civic initiatives to encourage patients’ empowerment | 4.1.1.127 | 382 | 0.30 | 0.55 | 0.08 | 0.16 | 0.56 | 152 |  |  |  |  |  |  |  |  |  |  | 230 |
| 1+ initiatives to inform citizens on conservation/donation of hematopoietic stem cells from cord blood | 4.1.1.128 | 227 |  |  |  |  |  | 39 |  |  |  |  | 26 |  |  |  |  |  | 162 |
| 1+ initiative to inform on procedures to express willingness to donate organs | 4.1.1.129 | 293 | 0.17 | 0.35 | 0.04 | 0.30 | 0.47 | 49 |  |  |  |  | 50 |  |  |  |  |  | 194 |
| Activities/initiatives of narrative medicine | 4.1.1.130 | 387 | 0.34 | 0.31 | -0.03 | 0.10 | 0.41 | 255 |  |  |  |  | 66 |  |  |  |  |  | 66 |
| 1+ training on cultural diversity for health prof. in contact with foreigners last 36 mts | 4.1.2.131 | 379 | 0.08 | 0.46 | 0.14 | 0.18 | 0.47 | 152 |  |  |  |  |  |  |  |  |  |  | 227 |
| 1+ training for clinicians on clinical comm and/or helping relationship last 36 mts | 4.1.2.132.1 | 386 | -0.09 | 0.86 | 0.08 | -0.06 | 0.44 | 59 |  |  |  |  |  |  |  |  |  |  | 327 |
| 1+ training for nurses on clinical comm. and/or helping relationship last 36 mts | 4.1.2.132.2 | 386 | -0.10 | 0.85 | 0.08 | -0.05 | 0.43 | 59 |  |  |  |  |  |  |  |  |  |  | 327 |
| 1+ training course on end-of-life management for healthcare professionals | 4.1.2.133 | 338 | 0.04 | 0.37 | 0.02 | 0.19 | 0.38 | 86 |  |  |  |  |  |  |  |  |  |  | 252 |
| 1+ survey on organizational climate and well-being over the last 24 mts | 4.1.2.134.1 | 386 | 0.21 | 0.22 | 0.02 | 0.12 | 0.35 | 114 |  |  |  |  |  |  |  |  |  |  | 272 |
| 1+ improvement action on organizational climate and well-being after the survey | 4.1.2.134.2 | 385 | 0.38 | 0.21 | 0.04 | 0.17 | 0.45 | 182 |  |  |  |  |  |  |  |  |  |  | 203 |
| Service Charter available at the facility | 4.2.1.135.1 | 387 | 0.12 | 0.03 | 0.44 | 0.37 | 0.50 | 80 |  |  |  |  |  |  |  |  |  |  | 307 |
| Service Charter available at the website | 4.2.1.135.2 | 387 | 0.16 | 0.09 | 0.74 | -0.01 | 0.47 | 27 |  |  |  |  |  |  |  |  |  |  | 360 |
| Service Charter updated maximum 36 months ago | 4.2.1.136.1 | 387 | 0.05 | 0.08 | 0.69 | 0.18 | 0.50 | 48 |  |  |  |  |  |  |  |  |  |  | 339 |
| Service Charter including general information on services and procedures to access and use services | 4.2.1.136.2 | 387 | 0.08 | 0.03 | 0.89 | 0.02 | 0.47 | 24 |  |  |  |  |  |  |  |  |  |  | 363 |
| Service Charter includes section on commitments with related evaluation tools | 4.2.1.136.3 | 387 | 0.23 | 0.06 | 0.40 | 0.32 | 0.51 | 134 |  |  |  |  |  |  |  |  |  |  | 253 |
| Service Charter includes section on protection from inefficiency and limitations | 4.2.1.136.4 | 387 | 0.21 | 0.21 | 0.58 | 0.13 | 0.56 | 64 |  |  |  |  |  |  |  |  |  |  | 323 |
| Qualitative/quantitative analysis of complaints at Patient and public relation and engagement Office last 24 months | 4.2.1.137.1 | 383 | 0.28 | 0.24 | 0.16 | 0.25 | 0.49 | 27 |  |  |  |  |  |  |  |  |  |  | 356 |
| 1+ actions/measures following analysis of complaints | 4.2.1.137.2 | 384 | 0.33 | 0.21 | 0.14 | 0.27 | 0.49 | 62 |  |  |  |  |  |  |  |  |  |  | 322 |
| Survey on user satisfaction delivered during the last 24 months | 4.2.1.138.1 | 384 | 0.54 | 0.01 | 0.30 | 0.06 | 0.46 | 97 |  |  |  |  |  |  |  |  |  |  | 287 |
| Public disclosure of Results of survey on user satisfaction | 4.2.1.138.2 | 384 | 0.68 | 0.01 | 0.16 | -0.02 | 0.41 | 218 |  |  |  |  |  |  |  |  |  |  | 166 |
| 1+ improvement actions implemented following the survey on user satisfaction | 4.2.1.138.3 | 383 | 0.69 | 0.04 | 0.17 | 0.17 | 0.52 | 171 |  |  |  |  |  |  |  |  |  |  | 212 |
| Service charter of birth clinical pathway services drafted | 4.2.1.139 | 226 |  |  |  |  |  | 72 |  |  |  |  |  |  |  |  |  |  | 154 |
| Information point in the hall at the main entrance of the facility | 4.2.2.140 | 387 | -0.06 | 0.09 | -0.05 | 0.56 | 0.32 | 67 |  |  |  |  |  |  |  |  |  |  | 320 |
| Welcome service | 4.2.2.141 | 387 | 0.04 | 0.12 | 0.03 | 0.50 | 0.39 | 157 |  |  |  |  |  |  |  |  |  |  | 230 |
| 1+ training courses on relation/communication to users for front staff last 36 months | 4.2.3.142 | 0.88 | 0.22 | 0.43 | 0.05 | 0.19 | 0.50 | 134 |  |  |  |  |  |  |  |  |  |  | 248 |
